# Effectiveness of a video-based smoking cessation intervention focusing on maternal and child health in promoting quitting among expectant fathers in China: A randomized controlled trial

**DOI:** 10.1371/journal.pmed.1003355

**Published:** 2020-09-29

**Authors:** Wei Xia, Ho Cheung William Li, Wenzhi Cai, Peige Song, Xiaoyu Zhou, Ka Wai Katherine Lam, Laurie Long Kwan Ho, Ankie Tan Cheung, Yuanhui Luo, Chunxian Zeng, Ka Yan Ho

**Affiliations:** 1 School of Nursing, University of Hong Kong, Hong Kong, China; 2 Shenzhen hospital, Southern Medical University, Shenzhen, China; 3 School of Public Health, Zhejiang University School of Medicine, Hangzhou, China; 4 Centre for Global Health Research, Usher Institute of Population Health Sciences and Informatics, The University of Edinburgh, Edinburgh, Scotland, United Kingdom; 5 Department of gynaecology and obstetrics, Nanhai Hospital Affiliated to Southern Medical University, Foshan, China; 6 School of Nursing, The Hong Kong Polytechnic University, Hong Kong, China; 7 Clinical Innovation & Research Center (CIRC), Shenzhen Hospital, Southern Medical University, Shenzhen, China; University of Stirling, UNITED KINGDOM

## Abstract

**Background:**

Secondhand smoke can cause adverse pregnancy outcomes, yet there is a lack of effective smoking cessation interventions targeted at expectant fathers. We examined the effectiveness of a video-based smoking cessation intervention focusing on maternal and child health in promoting quitting among expectant fathers.

**Methods and findings:**

A single-blind, 3-arm, randomized controlled trial was conducted at the obstetrics registration centers of 3 tertiary public hospitals in 3 major cities (Guangzhou, Shenzhen, and Foshan) in China. Smoking expectant fathers who registered with their pregnant partners were invited to participate in this study. Between 14 August 2017 to 28 February 2018, 1,023 participants were randomized to a video (n = 333), text (n = 322), or control (n = 368) group. The video and text groups received videos or text messages on the risks of smoking for maternal and child health via instant messaging. The control group received a leaflet with information on smoking cessation. Follow-up visits were conducted at 1 week and at 1, 3, and 6 months. The primary outcome, by intention to treat (ITT), was validated abstinence from smoking at the 6-month follow-up. The secondary outcomes included 7-day point prevalence of abstinence (PPA) and level of readiness to quit at each follow-up. The mean age of participants was 32 years, and about half of them were first-time expectant fathers. About two-thirds of participants had completed tertiary education. The response rate was 79.7% (815 of 1,023) at 6 months. The video and text groups had higher rates of validated abstinence than the control group (video group: 22.5% [75 of 333], P < 0.001; text group: 14.9% [48 of 322], P = 0.02; control group: 9.2% [34 of 368]) with adjusted odds ratios (ORs) of 2.80 (95% confidence interval [CI]: 1.79–4.37, P < 0.001) in the video group and 1.70 (95% CI: 1.06–2.74, P = 0.03) in the text group. The video and text groups differed in the rates of validated abstinence (22.5% versus 14.9%, P = 0.008; adjusted OR: 1.64, 95% CI: 1.10–2.46, P = 0.02). The video and text groups had higher rates of 7-day PPA than the control group at 6 months (video group: 24.6% [82 of 333] versus 11.4% [42 of 368], P < 0.001; text group: 17.4% [56 of 333] versus 11.4% [42 of 368], P = 0.02). The video and text groups also differed in the rates of 7-day PPA (24.6% versus 17.4%, P = 0.02). Excluding the quitters, the video and text groups had higher levels of readiness to quit than the control group at 6 months (video group: 43.5% [109 of 251] versus 31.6% [103 of 326], P = 0.002; text group: 40.6% [108 of 266] versus 31.6% [103 of 326], P = 0.01), No such difference was detected between the video and text groups (43.5% versus 40.6%, P = 0.29). The study was limited in that the long-term effectiveness of the intervention is uncertain.

**Conclusions:**

This smoking cessation intervention for expectant fathers that focused on explaining the ramifications of smoking on maternal and child health was effective and feasible in promoting quitting, and video messages were more effective than texts in delivering the information.

**Trial registration:**

ClinicalTrials.gov: NCT03236025.

## Abstract 2

Wei Xia and colleagues reveal the benefit of a video-based intervention to help expectant fathers quit smoking.

## Author summary

**Why was this study done?:**

- Exposure to secondhand smoke can cause adverse pregnancy and childbirth outcomes and negatively affect maternal and child health.
- Video has been used in multiple types of behavioral interventions, but there is a paucity of evidence on its effectiveness in changing addictive behaviors such as smoking.
- The aim of the study was to evaluate the effectiveness of a video-based smoking cessation intervention focusing on maternal and child health in promoting quitting among expectant fathers.

**What did the researchers do and find?:**

- A total of 1,023 smoking expectant fathers were randomly allocated to a video-based, text, or control group.
- Participants received four 1-minute videos (video group) or 4 text messages (text group) on the risks of smoking for maternal and child health via instant messaging every 2 weeks or a leaflet on smoking cessation (control group).
- At 6-month follow-up, participants who received the videos or texts had higher rates of validated abstinence than the controls. The video-based intervention was more effective than the text-based intervention.

**What do these findings mean?:**

- This trial provides evidence that videos incorporating information on the adverse effects of secondhand smoke on maternal and child health are effective in helping expectant fathers to quit smoking.
- Using instant messaging to deliver smoking cessation videos is feasible and acceptable to participants.

## Introduction

Exposure to secondhand smoke has been linked to adverse pregnancy and birth outcomes such as stillbirth, spontaneous abortion, tubal ectopic pregnancy, low birth weight, and fetal neurobehavioral developmental delays [1,2]. Newborns exposed to secondhand smoke have a higher risk of premature death, severe asthma, and slow lung growth [3]. A previous study reported that smoking outside the home and away from the infants or pregnant women (thirdhand smoke) does not completely protect a smoker’s home from environmental tobacco smoke contamination and infants or pregnant women from exposure to it [4]. It is therefore vital to help expectant fathers quit smoking, especially in China, where more than 40% of expectant fathers but only 3.8% of pregnant women smoke and approximately 75% of nonsmoking women reported regular exposure to secondhand smoke during their pregnancy [5,6]. Previous studies have indicated that shifts in masculinity associated with impending fatherhood may increase the eagerness of expectant or new fathers to participate in smoking cessation interventions [7,8].

A review of the literature found 2 trials to assist expectant fathers in quitting smoking [9,10]. In the first, 348 expectant fathers and their partners received couples counseling on smoking cessation and nicotine replacement therapy [9]. In the second, 48 couples received 15 minutes of counseling on smoking cessation [10]. However, neither trial found significant differences between the intervention and control groups. A systematic review of 9 qualitative studies published before 2014, summarizing the barriers to and facilitators of smoking cessation by male smokers during their partners’ pregnancy, found that most expectant fathers were aware of the adverse health effects of smoking but did not recognize that they extended to pregnant women and fetuses [11]. Innovative interventions that can effectively communicate the adverse effect of smoking on maternal and child health are therefore essential in helping expectant fathers quit smoking.

In China, leaflets containing information on smoking cessation are most commonly used by healthcare professionals. However, evidence shows that printed materials alone may be inefficient and are unlikely to change smokers’ behavior [12]. Busy clinical environments and a lack of training in smoking cessation often preclude healthcare professionals from delivering in-person, comprehensive, and specific health-related smoking cessation advice to expectant fathers who smoke [13].

Video-based intervention has been increasingly used in health promotion, including programs targeting breast self-examination, prostate cancer screening, treatment adherence, and female condom use [14]. This approach can be used as an alternative strategy to promote smoking cessation [15]. Video can be used to deliver health-related messages easily and effectively by providing auditory, visual, and verbal stimulation [16]. The use of sound and images can elicit emotions, better comprehension of abstract concepts, and improved retention of new information [17]. A systematic review of real-time video counseling for smoking cessation found no statistically significant treatment effects from telephone counseling, perhaps because of a paucity of high-quality trials and a lack of placebo controls [18]. In the present trial, we examined the effectiveness of a video-based smoking cessation intervention focusing on maternal and child health in promoting quitting among expectant fathers in China. We hypothesized that video-based intervention would be more effective than text messages and printed materials in increasing smoking cessation and that information focused on maternal and child health would be more effective than generic information in promoting smoking cessation among expectant fathers.

### Theoretical framework

The proposed study was guided by the theory of planned behavior, which emphasizes that intention is an important factor in determining behavior change [19]. This theory posits that 3 main factors affect intention: attitudes, subjective norms (individuals’ perceptions of others’ expectations for their behavioral change), and perceived behavioral control (individuals’ perceptions of their ability to change a behavior). Those individuals who have positive attitudes, who believe they have the approval of others, and who perceive themselves as capable of performing or controlling a behavior are motivated to change their behavior. The present intervention was designed to assist smoking expectant fathers in quitting smoking by changing their attitudes and subjective norms. We anticipated that providing information about smoking hazards during pregnancy and postpartum could change smoking expectant fathers’ attitudes regarding tobacco use and increase their awareness of how others around them (e.g., their pregnant partners) view their smoking behavior. Using video as a medium to convey information, we expected that smoking expectant fathers who received the intervention would increase their understanding of smoking hazards during pregnancy and postpartum and hence become more motivated to quit smoking. Because of the shifts in masculinity associated with impending fatherhood, expectant fathers’ responsibilities as providers and role models for children increases, which may increase their willingness to quit smoking [8].

## Methods

### Study design

A single-blind, multicenter, 3-arm, randomized controlled trial was conducted at the obstetrics registration centers of 3 tertiary public hospitals in 3 major cities (Guangzhou, Shenzhen, and Foshan) in China. This study followed the Consolidated Standards of Reporting Trials (CONSORT) 2010 guidelines (S1 CONSORT Checklist). Ethical approval for the study was obtained from the Institutional Review Board of The University of Hong Kong/Hospital Authority Hong Kong West Cluster (UW 17–269) and Shenzhen Hospital, Southern Medical University (NYSZYYEC20170017). The trial protocol is posted as S1 Text.

### Participants and settings

In China, pregnant women register for periodic prenatal examinations at obstetrics and gynecology clinics during weeks 8–12 of pregnancy. In general, expectant fathers are highly encouraged to attend the first visit with their partners. Expectant fathers were invited by research nurses to participate in the study if they met the following inclusion criteria: (1) aged 18 years or above, (2) had smoked at least 1 cigarette per day in the previous month and with a carbon monoxide level in expired air of 4 parts per million (ppm) or above, (3) able to read Chinese and communicate in Mandarin, and (4) able to use an instant messaging tool such as WeChat or WhatsApp for communication. Expectant fathers were excluded if they were unable to provide informed consent or receive counseling because of impaired mental status, cognitive impairment, or communication barriers identified from their medical records or if they had participated in other smoking cessation programs or services. Written consent was obtained from eligible expectant fathers after the purpose of the study was explained. Participants were also informed that their participation was voluntary.

### Randomization and masking

Each participant from the 3 hospitals was allocated into one of 3 groups by simple randomization. A random code list was generated using a personal computer by an independent research assistant with no involvement in participant recruitment. All participants were asked to scan the QR code for WeChat to connect with an intervention manager. The intervention manager then followed the order of the WeChat connection list to match the random codes with participants in chronological order of recruitment. This ensured concealment of group allocation. Single blinding was used, and research nurses collecting the data were blinded to the intervention allocation of participants. During the study period, participants could raise questions related to interventions received via WeChat, with minimal responses given by the intervention manager.

### Interventions

At baseline, all participants received brief advice on smoking cessation by research nurses: “Smoking is a high-risk activity that can be detrimental to your health. It would be better for you to quit smoking now.” In addition, participants received a leaflet published by the Chinese Center for Health Education and containing generic information on smoking cessation.

#### Video group

Participants received 4 videos on various risks of smoking for maternal and child health via WeChat. To increase the sustainability of the intervention, 1 video was sent to each participant in weeks 1, 3, 5, and 7.

Video content was developed using the theory of planned behavior. To ensure that the intervention had an adequate effect on the outcome measures of this study, a research committee was formed to assess the content in terms of the amount, frequency, duration, and breadth. The committee comprised an associate professor and an assistant professor with experience in smoking cessation interventions and an obstetrics nurse practitioner. Each video lasted approximately 1 minute, with content focusing on different hazardous effects of smoking on pregnant women, fetuses, and newborns (S2 Text). Additionally, to assess the suitability and acceptability of the proposed intervention to smoking expectant fathers and the feasibility of implementation in the clinics, a pilot study was conducted with 23 smoking expectant fathers in the obstetrics and gynecology clinics of the 3 hospitals during 1 July 2017 and 21 July 2017. The majority of expectant fathers commented that the content of the intervention was easy to comprehend and that they could view the content at their convenience. They also mentioned that the content was comprehensive and increased their awareness of the hazards of continued smoking to their health and the health of their pregnant partners and unborn children. Most importantly, we found that the intervention did not require much implementation time and thus was feasible in clinics despite their busy environment. Based on the positive results from the pilot study, the research committee recommended no change to the content of the proposed intervention. All expectant fathers who participated in the pilot study were excluded from the present trial.

#### Text group

Participants received 4 text messages with content similar to that of the videos and on similar schedules (S2 Text).

#### Control group

Following receipt of the leaflet at baseline, participants received no further intervention.

### Measures

#### Baseline measures

Participants’ baseline data, including demographic characteristics, health status, and smoking profiles, were obtained using a structured questionnaire (S3 Text) based on previous trials [20, 21]. The questionnaire was administered in person by the research nurses prior to randomization.

#### Outcome measures

Follow-up telephone calls were conducted at 1 week and 1, 3, and 6 months (S4 Text). The primary outcome was the validated 7-day point prevalence of abstinence (7-day PPA) at 6 months, confirmed by a carbon monoxide level in expired air of less than 4 ppm. This was originally a secondary outcome. We changed it to the primary outcome in October 2017 following a suggestion by a senior professor on the smoking cessation team who had no involvement in this study. The reason for the change was based on the Russell standard, in which biochemical validation of self-reported abstinence should be performed to ensure the consistency of reporting and allowed direct comparisons of these findings with those of other smoking cessation trials [22,23]. The change was implemented before the 6-month follow-up data were processed, with no effect on trial implementation.

Secondary outcomes were (1) self-reported 7-day PPA (the original primary outcome) and (2) levels of readiness to quit at 6 months.

#### Instruments

A structured questionnaire was developed by adapting international and nationally validated instruments. Participants’ health-related quality of life was assessed using the Chinese version of the 12-item Short-Form Survey for physical and mental health status, which showed good internal consistency (0.91) and reliability (0.81) [24]. Nicotine dependence was assessed using the Fagerström Test for Nicotine Dependence; the internal consistency for the Chinese version is 0.74 [25]. Smoking self-efficacy was assessed using the Smoking Self-Efficacy Questionnaire. The Chinese scale demonstrated excellent test–retest reliability, with 0.95 and 0.93 for internal and external stimuli, respectively [26].

### Statistical methods

Given that there were no data from similar trials, the required sample size was calculated according to a previous trial on the effectiveness of a text messaging intervention for smoking cessation among adult smokers, in which abstinence validated by exhaled carbon monoxide was 10.7% (268 of 2,735) in the intervention group and 4.9% (124 of 2,789) in the control group at 6 months [27]. To detect significant differences between the 3 groups with a power of 0.8, a one-sided significance level of 5% for the chi-squared test, and an allocation ratio of 1:1:1, 264 participants were required in each group. Given an expected dropout rate of 20% at the 6-month follow-up, we needed to recruit at least 990 participants.

We used the R programming language for statistical computing and graphics (version 3.1) to perform all data analyses. Descriptive statistics were used to calculate the frequency and percentage for categorical data or the mean and standard deviation for continuous data. Differences in demographic and smoking characteristics between the 3 groups were compared using the chi-squared test for categorical variables and analysis of variance for continuous variables [28]. Following the Russell standard, intention-to-treat (ITT) analysis was performed in the primary analysis, in which all participants were included in the analysis with the assumption that participants lost to follow-up were smokers with unchanged smoking habits. (Text A in S5 Text).

Univariable logistic regression was performed to obtain crude odds ratios (ORs) for the association between the intervention and primary or secondary outcomes. Generalized estimating equations (GEEs) were used to evaluate the effect of the intervention on the primary and secondary outcomes at each follow-up point with adjustment for age, hospital, employment status, annual income level, first-time expectant father, level of nicotine dependence, level of readiness to quit, and smoking self-efficacy at baseline. Repeated measures for estimating secondary outcomes considering the within-subject effect were also performed (Text B in S5 Text).

Subgroup analyses were performed by including multiplicative interaction terms in the equations. First-time expectant father, level of nicotine dependence, and readiness to quit within 30 days were included. A sensitivity analysis was conducted to compare the changes in adjusted estimates of primary and secondary outcomes using a pattern-mixture model with multiple imputation (PMM-MI) to handle missing data and completed cases excluding the missing values (Text C in S5 Text) [29].

## Results

**Fig 1:**
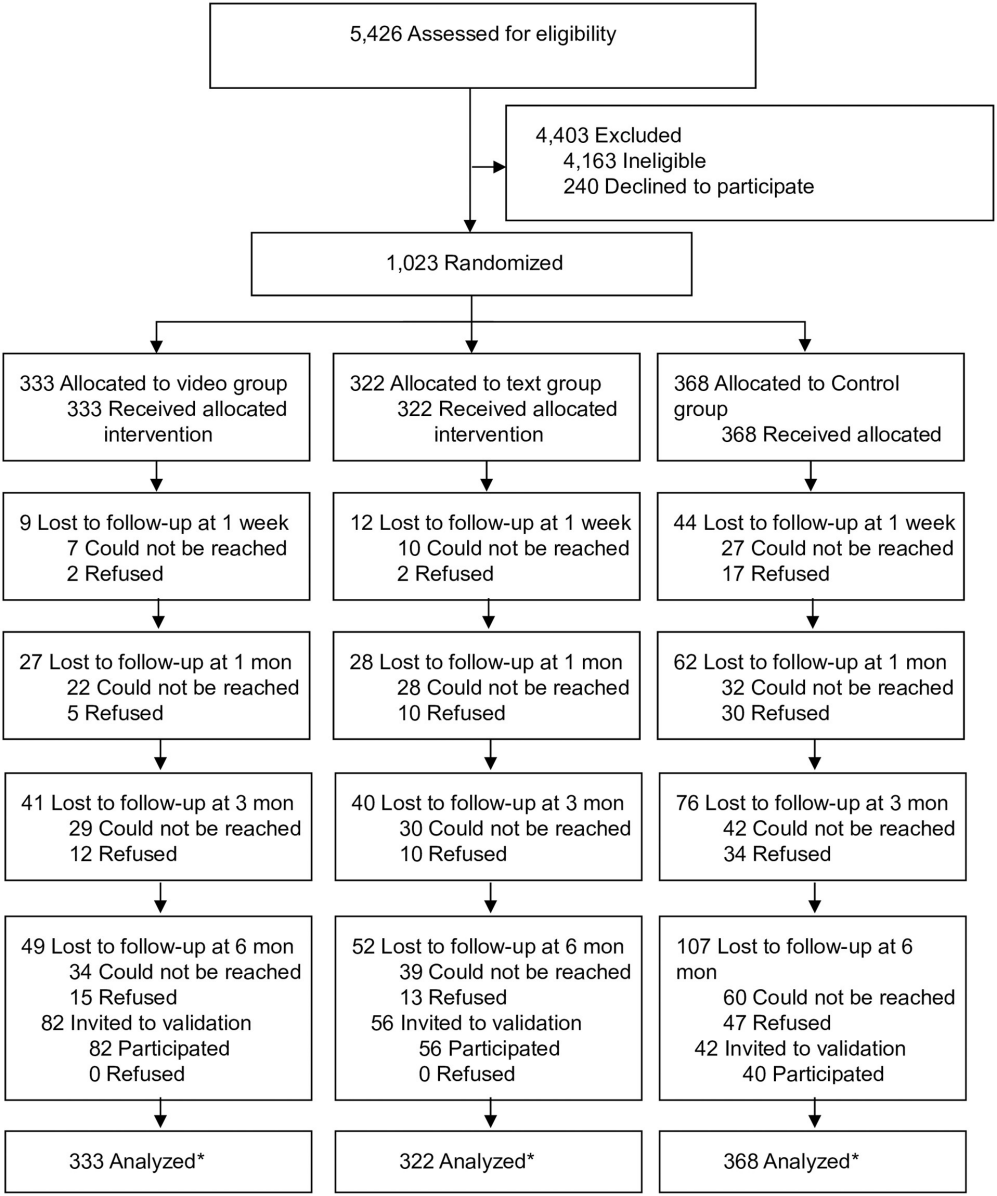
CONSORT flow diagram of participants through trial. Participants who were lost to follow-up previously were contacted again in the next follow-up. *An ITT analysis was performed by included all cases with the assumption that all participants lost to follow-up were smokers with unchanged smoking habits. CONSORT, Consolidated Standards of Reporting Trials; ITT, intention to treat.

From 14 August 2017 to 28 February 2018, we screened 5,426 expectant fathers. Of 1,263 eligible expectant fathers, 1,023 (80.8%) were willing to participate in the study. These 1,023 participants were randomly allocated to the video group (n = 333), text group (n = 322), or control group (n = 368). A total of 208 participants had withdrawn from the trial by the 6-month follow-up, with a retention rate of 79.7%. Of the 180 participants who self-reported quitting smoking at 6 months, 178 (98.9%) completed the exhaled CO validation test. The CONSORT flowchart is shown in Fig 1. The demographic characteristics and smoking profiles of participants among the 3 groups at baseline are presented in Table 1.

**Table 1:** Participants’ baseline demographic characteristics and smoking profiles.

Variables	No. (%)^a^		
	Video (n = 333)	Text (n = 322)	Control (n = 368)
Age, mean (SD), year	31.6 (5.5)	31.9 (5.3)	32.5 (5.2)
Employment status			
Unemployed	43 (14.5)	32 (11.8)	35 (15.0)
Self-employment	46 (13.5)	36 (10.5)	52 (10.1)
Employed	229 (72.0)	237 (77.7)	260 (74.9)
Education level			
Primary school or below	5 (1.5)	7 (2.2)	5 (1.4)
Middle school	111 (33.6)	99 (31.0)	95 (26.0)
College/university or above	214 (64.8)	213 (66.8)	265 (72.6)
Annual family income (CNY)^b^			
¥9,999 or below	44 (13.5)	37 (11.7)	56 (15.6)
¥10,000–49,999	47 (14.5)	67 (21.2)	53 (14.7)
¥50,000–99,999	110 (33.8)	99 (31.3)	121 (33.6)
¥100,000–199,999	68 (20.9)	52 (16.5)	52 (14.4)
¥200,000 or above	56 (17.2)	61 (19.3)	78 (21.7)
First-time expectant father			
Yes	178 (53.5)	176 (54.7)	180 (48.9)
No	155 (46.5)	146 (45.3)	188 (51.1)
Living with smoking partners			
Yes	2 (0.6)	0 (0)	3 (0.8)
No	331 (99.4)	322 (100)	365 (99.2)
Monthly regular alcohol use			
No	115 (34.5)	125 (38.8)	150 (40.8)
Yes	218 (65.5)	197 (61.2)	218 (59.2)
Regular activity at least 1 h/week			
Yes	109 (32.7)	106 (32.9)	123 (33.4)
No	224 (67.3)	216 (67.1)	245 (66.6)
Physical health status (SF-12-PCS), mean (SD)	52.6 (3.9)	53.2 (4.0)	53.0 (4.1)
Mental health status (SF-12-MCS), mean (SD)	52.4 (6.6)	52.8 (6.8)	53.5 (6.7)
Daily cigarette consumption, mean (SD)	9.7 (6.1)	9.9 (6.3)	9.0 (5.8)
Level of daily cigarette consumption			
Less than 10	242 (72.7)	234 (72.6)	276 (75.0)
11–20	84 (25.2)	81 (25.2)	90 (24.4)
More than 21	7 (2.1)	7 (2.2)	2 (0.5)
Dual use of e-cigarette	2 (0.6)	1 (0.3)	2 (0.5)
Dual use of IQOS	0 (0)	2 (0.6)	0 (0)
Nicotine dependence by FTND			
Low dependence (0–3 Fagerström score)	219 (66.4)	209 (65.1)	245 (66.9)
Moderate dependence (4–5 Fagerström score)	75 (22.7)	84 (26.2)	82 (22.4)
High dependence (6–10 Fagerström score)	36 (10.9)	28 (8.7)	39 (10.7)
Readiness to quit within 30 days			
Yes	35 (10.5)	23 (7.1)	34 (9.2)
No	298 (89.5)	299 (92.9)	334 (90.8)
Years of regular tobacco use	14.1 (6.4)	14.1 (5.5)	14.4 (5.9)
Quit attempt for 24 hours within 1 year			
Yes	73 (21.9)	83 (25.8)	95 (25.8)
No	260 (78.1)	239 (74.2)	273 (74.2)
Latest quit attempt date			
Within last month	8 (11.0)	9 (10.8)	14 (14.7)
More than 1 month ago	65 (89.0)	74 (89.2)	81 (85.3)
Looking for smoking cessation service			
Yes	0 (0.0)	1 (0.3)	0 (0.0)
No	333 (100.0)	321 (99.7)	368 (100.0)
Smoking self-efficacy scored by SEQ-12 (12–60), mean (SD)	32.0 (10.9)	30.7 (10.4)	32.0 (10.5)

**Abbreviations**: FTND, Fagerström Test of Nicotine Dependence; IQOS, “I quit original smoking” (the name of a new heated tobacco product); MCS, Mental Health Status; PCS, Physical Health Status; SEQ-12, Smoking Self-efficacy Questionnaire; SF-12, 12-Item Short-Form Survey.

^a^Sample sizes varied because of missing data on some variables.

^b^¥/CNY represents China Yuan; US$1.00 = ¥6.7.

### Primary outcomes

According to the ITT analysis, the video group showed higher proportions of validated abstinence at 6 months than the control group (22.5% [75 of 333] versus 9.2% [34 of 368], P < 0.001; Table 2). The text group similarly showed higher proportions of validated abstinence at 6 months than the control group (14.9% [48 of 322] versus 9.2% [34 of 368], P = 0.02). The GEE analysis showed that the video and text groups had significantly higher validated abstinence than the control group (adjusted OR for video group: 2.80, 95% confidence interval [CI]: 1.79–4.37, P < 0.001; adjusted OR for text group: 1.70, 95% CI: 1.06–2.74, P = 0.03, Table 3). Additionally, the video group had significantly higher validated abstinence than the text group (22.5% [75 of 333] versus 14.9% [48 of 322], P = 0.008), with an adjusted OR of 1.64 (95% CI, 1.10–2.46, P = 0.02) at 6 months. The number needed to treat was 7.5 for participants in the video group, much smaller than the 17.5 calculated for the text group.

**Table 2:** Smoking cessation outcomes of participants in the video, text, and control groups.

	No./Total No. (%)			P-Value for Video versus Text	P-Value for Video versus Control	P-Value for Text versus Control
Variables	Video (n = 333)	Text (n = 322)	Control (n = 368)			
Biochemically validated abstinence at follow-up						
6 months	75/333 (22.5)	48/322 (14.9)	34/368 (9.2)	0.008	<0.001	0.02
Self-reported 7-day PPA						
1 week	8/333 (2.4)	9/322 (2.8)	7/368 (1.9)	0.47	0.42	0.30
1 month	31/333 (9.3)	22/322 (6.8)	20/368 (5.4)	0.15	0.03	0.27
3 months	52/333 (21.6)	36/322 (11.2)	33/368 (9.0)	0.06	0.005	0.20
6 months	82/333 (24.6)	56/322 (17.4)	42/368 (11.4)	0.02	<0.001	0.02
Readiness to quit within 30 days^a^						
1 week	82/325 (25.3)	56/313 (17.9)	91/361 (25.2)	0.02	0.53	0.01
1 month	94/302 (31.1)	79/300 (26.3)	94/348 (27.0)	0.11	0.14	0.46
3 months	151/281 (53.7)	130/286 (45.5)	129/335 (38.5)	0.03	<0.001	0.05
6 months	109/251 (43.5)	108/266 (40.6)	103/326 (31.6)	0.29	0.002	0.01

^a^Quitters were excluded.

**Abbreviations**: PPA, point prevalence of abstinence

**Table 3:** GEE model for smoking cessation outcomes among 1,023 participants.

	Video versus Text				Video versus Control				Text versus Control			
Variables	Crude OR^a^ (95% CI)^a^	P-value	Adjusted OR^b^ (95% CI)^b^	P-value	Crude OR^a^ (95% CI)^a^	P-value	Adjusted OR^b^ (95% CI)^b^	P-value	Crude OR^a^ (95% CI)^a^	P-value	Adjusted OR^b^ (95% CI)^b^	P-value
Biochemically validated abstinence													
6 months	1.66 (1.11–2.47)	0.01	1.64 (1.10–2.46)	0.02	2.86 (1.85–4.42)	<0.001	2.80 (1.79–4.37)	<0.001	1.72 (1.08–2.75)	0.02	1.70 (1.06–2.74)	0.03
Self-reported 7-day PPA												
1 week	0.86 (0.33–2.25)	0.75	0.66 (0.25–1.73)	0.40	1.27 (0.46–3.54)	0.65	1.27 (0.45–3.58)	0.65	1.48 (0.55–4.03)	0.44	1.58 (0.53–4.74)	0.42
1 month	1.40 (0.79–2.47)	0.25	1.43 (0.81–2.50)	0.22	1.79 (1.00–3.20)	0.05	1.79 (0.99–3.22)	0.05	1.28 (0.68–2.38)	0.45	1.28 (0.68–2.39)	0.44
3 months	1.47 (0.93–2.32)	0.10	1.48 (0.94–2.33)	0.10	1.88 (1.18–2.99)	0.008	1.79 (1.12–2.86)	0.02	1.28 (0.78–2.10)	0.34	1.19 (0.71–1.99)	0.51
6 months	1.55 (1.06–2.27)	0.02	1.56 (1.07–2.29)	0.02	2.54 (1.69–3.81)	<0.001	2.50 (1.65–3.80)	<0.001	1.63 (1.06–2.52)	0.03	1.61 (1.04–2.50)	0.03
Readiness to quit within 30 days^c^												
1 week	1.55 (0.75–2.27)	0.07	1.52 (0.93–2.48)	0.09	1.01 (0.71–1.41)	0.99	0.81 (0.53–1.25)	0.32	0.65 (0.45–0.94)	0.02	0.53 (0.34–0.85)	0.008
1 month	1.34 (0.93–1.91)	0.11	1.33 (0.90–1.96)	0.16	1.26 (0.89–1.77)	0.19	1.13 (0.79–1.62)	0.51	0.94 (0.66–1.34)	0.74	0.87 (0.59–1.29)	0.49
3 months	1.48 (1.06–2.06)	0.02	1.56 (1.10–2.23)	0.01	1.83 (1.32–2.52)	<0.001	1.86 (1.33–2.61)	<0.001	1.24 (0.89–1.71)	0.20	1.23 (0.87–1.73)	0.23
6 months	1.07 (0.75–1.52)	0.71	1.04 (0.72–1.51)	0.84	1.71 (1.20–2.40)	0.003	1.70(1.18–2.43)	0.004	1.59 (1.13–2.23)	0.008	1.65 (1.15–2.36)	0.007

**Abbreviations**: CI, confidence interval; GEE, generalized estimating equation; OR, odds ratio; PPA, point prevalence of abstinence.

^a^Crude estimates from the univariable logistic regression

^b^Participants lost to follow-up were assumed to be active smokers with no changes in their habits at baseline. Estimates from the GEE model adjusted for age, hospital, employment status, annual income level, hospital, first-time expectant father, level of nicotine dependence, level of readiness to quit, and smoking self-efficacy at baseline.

^c^Quitters were excluded.

### Secondary outcomes

The 7-day PPA in the video and text groups was statistically significantly higher than that in the control group at 6 months (video group: 25.7% [82 of 333] versus 11.4% [42 of 368], P < 0.001; text group: 17.4% [56 of 333] versus 11.4% [42 of 368], P = 0.02). GEE analysis found that adjusted ORs were higher in the video intervention group (2.50, 95% CI: 1.65–3.80, P < 0.001) and text group (1.61, 95% CI: 1.04–2.50, P = 0.03) than in the control group. The video group also had significantly higher 7-day PPA than the text group (24.6% [82 of 333] versus 17.4% [56 of 322], P = 0.02), with an adjusted OR of 1.56 (95% CI: 1.07–2.29, P = 0.02) at 6 months. Excluding the quitters, the proportions of expectant fathers who were ready to quit within 30 days in the video and text groups were higher than in the control group at 6 months (video group: 43.5% [109 of 251] versus 31.6% [103 of 326], P = 0.002; text group: 40.6% [108 of 266] versus 31.6% [103 of 326], P = 0.01), with an adjusted OR of 1.70 (95% CI: 1.18–2.43], P = 0.004) in the video group and 1.65 (95% CI: 1.15–2.36, P = 0.007) in the text group. No such difference was detected between the video and text groups (43.5% [109 of 251] versus 40.6% [108 of 266], P = 0.29), with an adjusted OR of 1.04 (95% CI: 0.72–1.51, P = 0.84). S1 Table present the results of repeated measures. S1A and S1B Fig show the changes of secondary outcomes with the follow-ups.

### Subgroup analysis

The adjusted OR for the intervention effects on smokers who were first-time expectant fathers was similar to ORs of expectant fathers who had at least 1 child (Table 4, S6 Text). The adjusted ORs for the intervention effects were significantly higher for fathers with low levels of nicotine dependence than for those with moderate to high levels of nicotine dependence (video group with low dependence—adjusted OR: 4.53, 95% CI: 2.47–8.01; moderate/high dependence—adjusted OR: 1.49, 95% CI: 0.67–3.32; interaction P = 0.01. Text group with low dependence—adjusted OR: 2.82, 95% CI: 1.56–5.13; moderate/high dependence—adjusted OR: 1.10, 95% CI: 0.61–1.53; interaction P = 0.03). Among fathers who were not ready to quit within 30 days, the adjusted OR for the intervention effect was significantly higher than among fathers ready to quit within 30 days (video group members ready to quit within 30 days—adjusted OR: 2.29, 95% CI: 0.51–10.63; not ready to quit within 30 days—adjusted OR: 3.91, 95% CI: 2.20–6.92; interaction P = 0.03. Text group members ready to quit within 30 days—adjusted OR: 0.90, 95% CI: 0.22–4.10; not ready to quit within 30 days—adjusted OR: 2.29, 95% CI: 1.22–4.00; interaction P = 0.03). No differences were identified between the video and text groups in subgroup comparisons of first-time expectant fathers, nicotine dependence level, or the level of readiness to quit within 30 days.

**Table 4:** The validated abstinence at 6 months by subgroups.

	No./Total No. (%)			Video versus Text Adjusted OR (95% CI)^a^	P-Value for Interaction	Video versus Control Adjusted OR (95% CI)^a^	P-Value for Interaction	Text versus Control Adjusted OR (95% CI)^a^	P-Value for Interaction
	Video (n = 333)	Text (n = 322)	Control (n = 368)						
First-time expectant father					0.45		0.99		0.92
Yes	42/178 (23.6%)	29/176 (16.5%)	18/180 (10.0%)	1.60 (1.07–2.80)		2.57 (1.34–4.94)		1.77 (0.92–3.40)	
No	33/155 (21.3%)	19/146 (13.0%)	16/188 (8.5%)	1.82 (1.05–3.45)		3.18 (1.64–6.12)		1.90 (0.88–4.10)	
Nicotine dependence level (FTND)					0.21		0.01		0.03
Low (0–3)	54/219 (24.7%)	35/209 (16.7%)	17/245 (6.9%)	1.72 (1.05–2.83)		4.53 (2.47–8.01)		2.82 (1.56–5.13)	
Moderate/high (4–10)	21/111 (18.9%)	13/112 (11.6%)	16/121 (13.2%)	1.76 (0.89–3.57)		1.49 (0.67–3.32)		1.10 (0.61–1.53)	
Readiness to quit within 30 days					0.17		0.03		0.03
Yes	8/35 (22.9)	3/23 (13.0)	6/34 (17.6)	2.88 (0.32–24.20)		2.29 (0.51–10.63)		0.90 (0.22–4.10)	
No	67/298 (22.5)	45/299 (15.1)	28/334 (8.4)	1.50 (1.06–2.32)		3.91 (2.20–6.92)		2.29 (1.22–4.00)	

**Abbreviation:** CI, confidence interval; FTND, Fagerström Test of Nicotine Dependence; GEE, generalized estimating equation; OR, odds ratio.

^a^Subjects lost to follow-up were assumed to be active smokers with no changes in their habits at baseline. Estimates from the GEE model adjusted for age, hospital, employment status, annual income level, hospital, first-time expectant father, level of nicotine dependence, level of readiness to quit, and smoking self-efficacy at baseline.

### Sensitivity analysis

No significant differences were identified in the 3 groups between participants who completed the trials and those who withdrew, with the exception of dropout numbers (14.7% [49 of 333] in the video group versus 16.1% [52 of 322] in the text group versus 29.1% [107 of 368] in the control group, P < 0.001) and quit attempts within the preceding year (26.0% [212 of 815] versus 18.8% [39 of 208], P = 0.02; S2 Table). A comparison of the results obtained using ITT analysis, PMM-MI, and completed cases showed similar parameter estimates (Table 5).

**Table 5:** The sensitivity analysis for primary and secondary outcomes by using PMM-MI and completed case.

	Video versus Text				Video versus Control				Text versus Control			
	PMM-MI^b^ Adjusted OR (95% CI)^d^	P-Value	CC^c^ Adjusted OR (95% CI)^d^	P-Value	PMM-MI^b^ Adjusted OR (95% CI)^d^	P-Value	CC^c^ Adjusted OR (95% CI)^d^	P-Value	PMM-MI^b^ Adjusted OR (95% CI)^d^	P-Value	CC^c^ Adjusted OR (95% CI)^d^	P-Value
Biochemically validated abstinence												
6 months	1.66 (1.11–2.47)	0.01	1.58 (1.03–2.43)	0.03	2.80 (1.80–4.38)	<0.001	2.77 (1.80–4.28)	<0.001	1.71 (1.06–2.74)	0.01	1.67 (1.05–2.65)	0.03
Self-reported 7-day PPA												
1 week	0.71 (0.26–1.92)	0.49	0.63 (0.22–1.84)	0.40	1.21 (0.42–3.51)	0.73	1.10 (0.34–3.53)	0.87	1.44 (0.47–4.41)	0.52	1.76 (0.60–5.11)	0.30
1 month	1.36 (0.77–2.41)	0.28	1.26 (0.70–2.28)	0.44	1.69 (0.93–3.06)	0.09	1.61 (0.87–2.99)	0.13	1.26 (0.66–2.37)	0.48	1.29 (0.68–2.45)	0.43
3 months	1.39 (0.88–2.22)	0.16	1.32 (0.81–2.16)	0.27	1.73 (1.08–2.77)	0.02	1.50 (0.90–2.49)	0.12	1.22 (0.72–2.06)	0.46	1.16 (0.68–1.20)	0.59
6 months	1.60 (1.08–2.38)	0.02	1.50 (1.01–2.27)	0.04	2.03 (1.32–3.11)	0.001	1.99 (1.27–3.11)	0.003	1.27 (1.03–1.99)	0.03	1.33 (1.12–1.92)	0.03
Readiness to quit within 30 days^a^												
1 week	1.59 (1.07–2.38)	0.04	1.53 (1.04–2.26)	0.03	0.94 (0.67–1.34)	0.74	0.93 (0.66–1.33)	0.71	0.62 (0.42–0.90)	0.01	0.62 (0.42–0.91)	0.01
1 month	1.35 (0.92–1.99)	0.13	1.26 (0.87–1.81)	0.22	1.07 (0.74–1.53)	0.73	1.16 (0.82–1.63)	0.41	0.82 (0.56–1.21)	0.32	0.94 (0.66–1.35)	0.75
3 months	1.34 (0.90–1.99)	0.15	1.46 (1.02–2.15)	0.03	1.72 (1.18–2.49)	0.004	1.86 (1.28–2.68)	0.001	1.29 (0.88–1.89)	0.19	1.29 (0.89–1.86)	0.18
6 months	1.07 (0.71–1.62)	0.74	1.16 (0.81–1.67)	0.41	1.64 (1.13–2.88)	0.01	1.57 (1.10–1.91)	0.01	1.48 (1.12–1.84)	0.03	1.34 (1.06–1.91)	0.03

**Abbreviations:** CC, Completed case; CI, confidence interval; OR, odds ratio; PMM-MI, Pattern-Mixture Model with Multiple Imputation; PPA, point prevalence of abstinence.

^a^Quitters were excluded.

^b^Outcomes missing in each follow-up were imputed according to pattern specified by the identifying restriction (S2 Text).

^c^The missing data were ignored in the analysis.

^d^Estimates adjusted for age, hospital, employment status, annual income level, hospital, first-time expectant father, level of nicotine dependence, level of readiness to quit, and smoking self-efficacy at baseline

### Adverse events

No adverse events were reported during the entire study period.

## Discussion

This trial examined the effectiveness of a video-based intervention focusing on maternal and child health in helping expectant fathers to quit smoking. Overall, the video-based intervention was more effective than text messages and printed materials in promoting quitting among smoking expectant fathers at 6 months. The results also demonstrate the suitability of using video as a medium to deliver smoking cessation interventions. Apart from better comprehension of abstract concepts and better retention of new information through auditory, visual, and verbal stimulation, video-based intervention can be delivered via an easily accessible mobile device, on which content can be viewed at the participants’ convenience and at their own pace. Furthermore, video can communicate important messages to smokers in less time than it takes to read some texts. Video imagery can help the brain to create initial impressions supplemented with detailed and in-depth verbal descriptions, resulting in more sustainable effects on attempts at smoking cessation [30]. However, the superiority of video over text in this context requires a longer time to emerge and becomes more apparent at 6 months [31].

Participating fathers who received text messages on the adverse effects of smoking on maternal and child health had a significantly higher validated abstinence rate at 6 months than those who received a leaflet containing generic information on smoking cessation. These findings provide support to our argument that smoking cessation advice that includes information on the detrimental effects of smoking to the health of pregnant women, fetuses, and children is crucial in motivating expectant fathers to quit.

This study has some important strengths. First, the research question has been underexplored, according to the existing literature. Second, compared with previous trials of smoking cessation for expectant fathers [9,10], we recruited a large sample from multiple centers in several cities. We had a good retention rate of 79.7% and a high participation rate for biochemical validation (98.9%) at 6 months, which increases the generalizability of the findings. Third, we complied with the Russell standard to perform biochemical validation as the primary outcome assessment, which is the gold standard in smoking cessation trials [32].

### Limitations

This trial was limited in that we only assessed the outcomes up to 6 months. A longer follow-up is required to study the long-term effects of video-based intervention for smoking expectant fathers, particularly as they relate to postpartum relapse, which is common in this population [33,34]. Another limitation is that the dropout rate at 6 months in the control group (29.1%) was higher than the rate in the video (14.7%) and text (16.6%) groups. A higher attrition rate among controls is common in clinical trials, especially because we used a smaller intervention in the control group; thus, control-group participants may have concluded that the intervention was ineffective in helping them to quit smoking. To minimize the impact of missing values, PMM-MI was used to handle missing data, and sensitivity analyses were conducted [29]. Small discrepancies and similar direction and significance of all estimates observed between the results obtained from sensitivity analyses indicated that the findings supported the study hypotheses [35].

### Implications for clinical practice

The World Health Organization has emphasized that healthcare professionals play a prominent role in smoking cessation by providing advice, guidance, and responses to questions related to smoking and associated health issues [36]. Nevertheless, a lack of training in smoking cessation and busy clinical settings often make it impossible for healthcare professionals to deliver comprehensive in-person advice to help smokers quit. This trial demonstrates the effectiveness and suitability of a video-based intervention to help expectant fathers quit smoking, which can serve as a strategy to overcome barriers to the promotion of smoking cessation by healthcare professionals in clinical practice. One advantage of video-based intervention for smoking cessation is that a variety of videos can be created by healthcare professionals according to specific health-related issues, and these videos can be used repeatedly. Healthcare professionals can select and use appropriate videos with engaging content to help smokers quit. Innovative technology has facilitated the inexpensive production of videos and other necessary equipment, which are now available at low cost. The cost of compiling the 4 videos was approximately US$213, making video-based intervention a cost-effective and sustainable approach to helping smokers to quit. More importantly, in addition to smoking cessation, healthcare professionals can consider using video-based interventions to encourage healthy lifestyle habits such as good dietary choices and reduced alcohol consumption for the prevention and control of noncommunicable diseases.

### Conclusions

This trial demonstrated the effectiveness of a video-based intervention in promoting smoking cessation among expectant fathers. Healthcare professionals can further examine the long-term effectiveness of this simple, feasible, and low-cost strategy and consider its use to promote smoking cessation in clinical practice.

## Supporting information

S1 CONSORT Checklist CONSORT, Consolidated Standards of Reporting Trials. (DOCX)

S1 Text Research protocol. (PDF)

S2 Text Translated content of the interventional videos and text. (DOCX)

S3 Text Baseline structured questionnaire. (PDF)

S4 Text Follow-up questionnaires. (PDF)

S5 Text Details of statistical analysis method. (S5A) Multiple imputation. (S5B) GEE model. (S5C) Sensitivity analysis. GEE, generalized estimating equation
(DOCX)

S6 Text Finding in the subgroup analysis. (DOCX)

S1 Table Repeated measure for secondary outcomes. (DOCX)

S2 Table Demographic and smoking characteristics between participants who completed the study and those who were lost to follow-up at 6 months. (DOCX)

S1 Fig Changes of secondary outcomes with the follow-ups. (S1A) Comparison of self-reported 7-day PPA among the 3 groups. (S1B) Comparison of readiness to quit within 30 days among the 3 groups. PPA, point prevalence of abstinence.
(TIF)

## Abbreviations

CI: confidence interval
CONSORT: Consolidated Standards of Reporting Trials
GEE: generalized estimating equation
ITT: intention to treat
OR: odds ratio
PMM-MI: pattern-mixture model with multiple imputation
PPA: point prevalence of abstinence
ppm: parts per million
