# Supplementary material for: Effectiveness of a video-based smoking cessation intervention focusing on maternal and child health in promoting quitting among expectant fathers in China: A randomized controlled trial
Source: PLoS Med. 2020 Sep 29;17(9):e1003355. doi: 10.1371/journal.pmed.1003355 (PMC7523971; doi:10.1371/journal.pmed.1003355)
Supplement: S1 CONSORT Checklist — (DOCX) [file pmed.1003355.s001.docx]

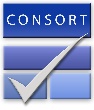
 S1. CONSORT Checklist

| Section/Topic | Item No | Checklist item | Reported on page No |
| --- | --- | --- | --- |
| Title and abstract | | | |
|  | 1a | Identification as a randomised trial in the title | Title |
|  | 1b | Structured summary of trial design, methods, results, and conclusions (for specific guidance see CONSORT for abstracts) | Abstract section |
| Introduction | | | |
| Background and objectives | 2a | Scientific background and explanation of rationale | Paragraph 1-4, Introduction section |
|  | 2b | Specific objectives or hypotheses | Paragraph 4, Introduction section |
| Methods | | | |
| Trial design | 3a | Description of trial design (such as parallel, factorial) including allocation ratio | Paragraph 1, Study design sub-section, Method section |
|  | 3b | Important changes to methods after trial commencement (such as eligibility criteria), with reasons | NA |
| Participants | 4a | Eligibility criteria for participants | Paragraph 1, Participants and settings sub-section, Method section |
|  | 4b | Settings and locations where the data were collected | Paragraph 1, Participants and settings sub-section, Method section |
| Interventions | 5 | The interventions for each group with sufficient details to allow replication, including how and when they were actually administered | Paragraph 1-5, Intervention sub-section, Method section |
| Outcomes | 6a | Completely defined pre-specified primary and secondary outcome measures, including how and when they were assessed | Paragraph 2-3, Measures sub-section, Method section |
|  | 6b | Any changes to trial outcomes after the trial commenced, with reasons | Paragraph 2, Measures sub-section, Method section |
| Sample size | 7a | How sample size was determined | Paragraph 1, Statistical methods sub-section, Method section |
|  | 7b | When applicable, explanation of any interim analyses and stopping guidelines | NA |
| Randomisation: |  |  |  |
| Sequence generation | 8a | Method used to generate the random allocation sequence | Paragraph 1, Randomization and masking sub-section, Method section |
|  | 8b | Type of randomisation; details of any restriction (such as blocking and block size) | Paragraph 1, Randomization and masking sub-section, Method section |
| Allocation concealment mechanism | 9 | Mechanism used to implement the random allocation sequence (such as sequentially numbered containers), describing any steps taken to conceal the sequence until interventions were assigned | Paragraph 1, Randomization and masking sub-section, Method section |
| Implementation | 10 | Who generated the random allocation sequence, who enrolled participants, and who assigned participants to interventions | Paragraph 1, Randomization and masking sub-section, Method section |
| Blinding | 11a | If done, who was blinded after assignment to interventions (for example, participants, care providers, those assessing outcomes) and how | Paragraph 1, Randomization and masking sub-section, Method section |
|  | 11b | If relevant, description of the similarity of interventions | NA |
| Statistical methods | 12a | Statistical methods used to compare groups for primary and secondary outcomes | Paragraph 2-4, Statistical methods sub-section, Method section |
|  | 12b | Methods for additional analyses, such as subgroup analyses and adjusted analyses | Paragraph 5, Statistical methods sub-section, Method section |
| Results | | | |
| Participant flow (a diagram is strongly recommended) | 13a | For each group, the numbers of participants who were randomly assigned, received intended treatment, and were analysed for the primary outcome | Paragraph 1, Results section, fig1 |
|  | 13b | For each group, losses and exclusions after randomisation, together with reasons | Paragraph 1, Results section, fig1 |
| Recruitment | 14a | Dates defining the periods of recruitment and follow-up | Paragraph 1, Results section, fig1 |
|  | 14b | Why the trial ended or was stopped | Paragraph 1, Results section, fig1 |
| Baseline data | 15 | A table showing baseline demographic and clinical characteristics for each group | Table 1 |
| Numbers analysed | 16 | For each group, number of participants (denominator) included in each analysis and whether the analysis was by original assigned groups | Fig1 |
| Outcomes and estimation | 17a | For each primary and secondary outcome, results for each group, and the estimated effect size and its precision (such as 95% confidence interval) | Paragraph 2-3, Results section, table 2 |
|  | 17b | For binary outcomes, presentation of both absolute and relative effect sizes is recommended | 13, table 3 |
| Ancillary analyses | 18 | Results of any other analyses performed, including subgroup analyses and adjusted analyses, distinguishing pre-specified from exploratory | Paragraph 4-5, Results section, fig1, table 4-5, S2 and S3 Table. S1 Figure |
| Harms | 19 | All important harms or unintended effects in each group (for specific guidance see CONSORT for harms) | Paragraph 6, Results section |
| Discussion | | | |
| Limitations | 20 | Trial limitations, addressing sources of potential bias, imprecision, and, if relevant, multiplicity of analyses | Paragraph 4, Discussion section |
| Generalisability | 21 | Generalisability (external validity, applicability) of the trial findings | Paragraph 1, Discussion section |
| Interpretation | 22 | Interpretation consistent with results, balancing benefits and harms, and considering other relevant evidence | Paragraph 1-3, Discussion section |
| Other information | | |  |
| Registration | 23 | Registration number and name of trial registry | Abstract section |
| Protocol | 24 | Where the full trial protocol can be accessed, if available | S1 Text |
| Funding | 25 | Sources of funding and other support (such as supply of drugs), role of funders | EM |

*We strongly recommend reading this statement in conjunction with the CONSORT 2010 Explanation and Elaboration for important clarifications on all the items. If relevant, we also recommend reading CONSORT extensions for cluster randomised trials, non-inferiority and equivalence trials, non-pharmacological treatments, herbal interventions, and pragmatic trials. Additional extensions are forthcoming: for those and for up to date references relevant to this checklist, see [www.consort-statement.org](http://www.consort-statement.org).
