# Supplementary material for: Effectiveness of a video-based smoking cessation intervention focusing on maternal and child health in promoting quitting among expectant fathers in China: A randomized controlled trial
Source: PLoS Med. 2020 Sep 29;17(9):e1003355. doi: 10.1371/journal.pmed.1003355 (PMC7523971; doi:10.1371/journal.pmed.1003355)
Supplement: S1 Text — (PDF) [file pmed.1003355.s002.pdf]

S1 Text. Research protocol

Title: The Effectiveness of a Video-led Smoking Cessation Intervention in Helping Male Smokers in Mainland China Whose Partner Got Pregnant to Quit: A Randomized Controlled Trial

S. 1. Background:

With a globally growing number of smokers, up to about half of men and one tenth of women are becoming smokers and relatively few stopping [1, 2]. The global smoking report presents that most of smokers live in middle- and low-income countries including China, where the availability of smoking cessation service is scarce [3].

There is evidence that tobacco is responsible for even more deaths and diseases [3]. The number of deaths attributed to tobacco use has reached 1.2 million per year, whereas the death toll is expected to rise to 2 million annually by 2025 [4]. Smokers have two to three times mortality in middle age among otherwise similar persons who had never smoked [3]. The smokers that still live in middle age also have lost at least a decade of life time, because the tobacco is also the biggest external cause of non-communicable disease [5]. According to the literature review, smoking has been confirmed to be an important risk factor to coronary heart disease, chronic obstructive lung disease, cancer, stroke, peptic ulcer, peripheral vascular disease, aortic aneurysm, osteoporosis, low bone density, cataract, pneumonia, periodontitis, subclinical arteriosclerosis[6, 7]. In addition, tobacco can also increase the incidence rate of cancer in lung, oral cavity, naso-, oro- and hypopharynx, nasal cavity and accessory sinuses, larynx, oesophagus, stomach, pancreasm colorectum, liver, kidney ureter, urinary bladder, uterine cervix and ovary, and myeloid leukemia, among smokers [8-10].

However, the impact caused by tobacco is far more than that. For the male smokers at their reproductive ages, who is preparing to have a baby, smoking may negatively influence their reproductive capacity. Sufficient studies found that the quantity and duration of smoking, especially the current smoking is positively associated with the risk of erectile dysfunction[11, 12]. The clinical study provided strong indirect evidence that smoking may affect penile erection by the impairment of endothelium dependent smooth muscle relaxation. The association of erectile dysfunction with risk factors such as coronary artery disease and hypertension appears to be amplified by cigarette smoking[13]. Besides, the quality and quantity of sperm of smokers were lower than that of non-smokers. Even though, the mechanism is not clear, many research results has reported that smokers had a significant decrease in the semen volume, and rapid progressive motility and sperm viability [14, 15]. Moreover, smokers had a significant increase in the levels of immotile sperms and semen leukocytes. Sperm motion parameters were all lower in the smokers [16]. Further, the percentage of abnormal morphology sperm was significantly more than their non-smoker counterparts[17]. What's worse, the sperm morphology was worse with increasing degree of smoking [14, 18]. All

stated above means that smoking may increase the risk of a higher risk of developing oligospermia, asthenozoospermia and teratozoospermia for man in the reproductive years[15], which may lead to the inefficiency of fertilization and even give birth to abnormal fetus. According to the official report, more than half of males are smokers in mainland China [4]. In addition, half of them have been smokers before 20 years old [16]. That means there are a large among of male smokers are under the hazard of smoking.

Excepting for the impact on reproductive male smokers their own, Secondhand Smoking (SHS) exposure also has further and longer negative effects on their female partners and their babies born in the future. A cross-countries study showed that median air nicotine concentration was 17 times higher in households with smokers compared with households without smokers. However, it is widely admitted that there is no risk-free level of exposure to the SHS. The non-smoking population is much more sensitive to the nicotine in the passive smoking than smokers [17, 18]. The SHS may increase the risk of respiratory infections, ear problem, immediate adverse effects on cardiovascular system, CHD, lung cancer and so on to adults [19]. For children, the passive smoking takes children a higher chance to get severe asthma, slow lung growth. Hence, children and women live with smokers may have a higher risk of premature death and disease [20]. Moreover, the harm of SHS to the unborn fetus and newborn babies of male smokers is worse, even lethal. When compared with non-smoking women with no SHS exposure, non-smoking women with SHS exposure reported to have more proportion of spontaneous abortion, stillbirth, tubal ectopic pregnancy, preterm birth, and high risk of very preterm birth increased with the duration of exposure [21-23]. Plenty of research found that ETS exposure was also reported to be associated with an increased risk of aplasia, in which low birth weight was reported mostly [24]. Besides, newborns whose mothers were exposed to secondhand smoke showed significantly lower scores in the habituation cluster and motor system cluster [25], as well as the delay of neurobehavioral development regardless of socio-demographic, obstetric and pediatric factors [26]. Conclusionly, smoking has direct and indirect negative effect to male smokers in reproductive ages themselves and their family members. Therefor, finding solutions for this problem is extremely urgent.

So far, quitting smoking is the only proven efficient routine to essentially solve this probem. Several studies consistently concluded that persons who began smoking in early adulthood but stopped before 40 years of age avoid more than 90% of the excess risk during their next few decades of life, as compared with those who continue to smoke, and even those who stop at 50 years of age avoid more than half the excess risk [27-29]. What's more, the exposure dose to pregnant women and children has been proven to be partly associated with the negative impact above [30].

A global statistics review showed that there has been widespread cessation in many high-income countries, however, quitting is uncommon for smokers in China [3]. Based on the official statistic figures in 2010, about one third of people in China were current smokers, and the number of young smokers is increasing [4]. While most current smokers (83.9%) report having no intention

to quit smoking [31]. On average, between 60% and 70% of Chinese men continue to smoke into middle age, which is different with the western countries. [32]. Another reports in 2005 and 2010, respectively, indicated that about 74% and 84% of smokers did not want to quit or never thought about quitting[33, 34]. Approximately 20% to 25% of smokers had attempted to quit at least once, but about half relapsed [4]. Noteworthily, a global comparison report shows that the estimated age-standardized prevalence of daily smoking among male smokers and female smokers in China is located in the highest and lowest deciles around the world, respectively [35]. According to the global statistic data, the secondhand smoking(SHS) exposure of non-smoking children and females is ranged from 17.3% to 73.1% inside home[36-38]. In China, this average rate was around 60%, the highest one has reached to 82.5% in large cities [4, 39, 40]. We can infer from the data above that a large amount of men is smoking and a great number of women are suffering from the damage of second hand smoking in China. Hence, finding out a solution to decrease the married reproductive male smoking rate is extremely crucial.

Based on surveys conducted in China, excepting for the sociocultural factors favouring smoking initiation, lack of awareness the hazards of smoking, and weak social support are mainly responsible for the low rate of smoking cessation for male individuals in China [41, 42].

Overall awareness of the health hazards of tobacco has improved in the last 15 years in China, but is still relatively poor [43].Yang and his colleagues found that over 80% of the males was aware that smoking causes serious diseases but over half of these people can not identify what kinds of disease can be caused by smoking. Less than two thirds of males were aware that secondhand smoke can cause serious diseases. Besides them, only small part of people considered that secondhand smoking can cause serious impact on children [43]. Even there were several patterns of propaganda and education applied in China, the efficiency is not very satisfactory. Lacking of motivation was the main explanation for the low smoking cessation rate. In China, parents' high attention on the children is general. Hence, emphasizing the health hazards of tobacco to male smokers and secondhand smoking to their wives and baby born in the future shoule probabliy be a effective motivation for reproductive male smokers in China.

Besides, belief in the ability to control the health effects of smoking is another barrier of smoking cessation for Chinese male smokers on the individual level [44]. Self-efficacy is the belief that one has the power to produce that effect by completing a given task or activity related to that competency [45, 46]. Generally, quitting smoking is well recognized to positively associated with the smoking cessation[47, 48]. It influence the smokers' thought patterns and emotional reactions. High self-efficacy helps create feelings of serenity in approaching difficult to quit smoking. Converging evidence from diverse methodological and analytic strategies verifies that perceived self-efficacy and personal goals enhance motivation and performance attainments[49]. When self-efficacy belief and outcome expectation differ, the self-efficacy belief is more likely to determine the behavior[50]. That means a high self-efficacy may indicate a high possibility to change to quit

and a less chance of relapse for smokers [47]. Thus, enhance the self-efficacy of changing for male smokers is a crucial steps to quitting smoking. Previous research found that, providing information and knowledge related to how to change can improve the self-efficacy of behavior changing[51]. By understanding how to change and coping with the negative physical and psychological reactions, the smokers will be more confident to the smoking cessation. Besides, social support, especially the family support, can also enhance the smokers' self-efficacy related to smoking cessation [52, 53]. By maintaining a positive regard and provide psychological decompression for the smoker, supportive family may motivates the smoker for quitting smoking[54]. Hence, improving the information and communication to increase the knowledge of the adverse impact of smoking on the smokers, family members and their baby born in the future to male smokers and their family members especially their wives, is an important way to enhance their motivation to quit smoking. Future smoking control initiatives that target non-smoking women to influence male smoking should take into account the women's overarching need to maintain the status and harmony of their families[55]. To sum up, useful information and family support can help to enhance the self-efficacy, and further promote the behavior of quitting smoking for male smokers[56].

Different patterns of intervnetions, such as counselling, advertising, leaflets and so on have been implemented, however the effect is not so well as we expected. Thus, a more efficient intervention should be developed. As the high usage rate of smartphone, intervnetions implemented by cellphone can be considered as a new approach. Watching video has been proven to be significantly associated with prevalence abstinence [53]. Feedback from participants indicated that the support provided by the video role models was important and appreciated. In a multiple intervention medium, the text is fast and precise, but it's also impersonal and monotonous to interpretation without tone; voice can express inflection and need less attention, but easy to miss information; images can stimulate the visual power and improve the delights, however, it has limits to delivery information. Video, combining with the advantage of text, image, and tones, will take visual, auditory and reading stimulation to the brain, consequently, enhance the delivery and memory of information for people. Hence video is an efficient intervention medium for smoking cessation[57].

Cigarette can negatively influence the function of respiratory, urinary and other systems, as well as increase the risk of different types of cancer. The secondhand smoking is harmful to their family members. What's worse, the impact of reproductive system will further damage their baby born in the future. For Chinese people, the high prevalence and low quitting rate made this situation much worse. In addition, as the cancel of one-child policy, lots of couples are planning to give birth to baby in the future decades compared with the period the policy was valid. Hence, an intervention should be urgent to be applied to help male whose partners got pregnant to quit or reduce the cigarette smoking. Hence this study aims to

(1) Measure the smoking consume of male smokers whose partner got pregnant,

(2) Developing a media-led intervention to help these male smokers whose partner got pregnant,

(3) To detect the effectiveness of the media-led intervention for the male smokers whose partner got pregnant.

S. 2. Theoretical framework:

A risk communication approach guided by the theory of planned behavior (TPB) will be used in this study. The TPB is a theory that links beliefs and behavior proposed by Icek Ajzen to improve on the predictive power of the theory of reasoned action by including perceived behavioural control[58]. It has been applied to studies of the relations among beliefs, attitudes, behavioral intentions and behaviors in various fields such as public relations, healthcare, as well as smoking cessation in resent years[59].

The theory states that attitude toward behavior, subjective norms, and perceived behavioral control, together shape an individual's behavioral intentions and behaviors. if people evaluate the suggested behavior as positive (attitude), and if they think their significant others want them to perform the behavior (subjective norm), this results in a higher intention (motivations) and they are more likely to do so. A high correlation of attitudes and subjective norms to behavioral intention, and subsequently to behavior, has been confirmed in many studies.

However, because of circumstantial limitations, behavioral intention does not always lead to actual behavior. Namely, since behavioral intention cannot be the exclusive determinant of behavior where an individual's control over the behavior is incomplete. Hence, perceived behavioral control was induced. Perceived behavioral control, refers to the degree to which a person believes that they control any given behavior (class notes). The theory of planned behavior suggests that people are much more likely to intend to enact certain behaviors when they feel that they can enact them successfully. Increased perceived behavioral control is a mix of two dimensions: self-efficacy and controllability. Self-efficacy, which was proposed by Bandura in 1977 in the social cognitive theory, refers to the level of difficulty that is required to perform the behavior, or one's belief in their own ability to succeed in performing the behavior. Controllability refers to the outside factors, and one's belief that they personally have control over the performance of the behavior, or if it is controlled by externally, uncontrollable factors. If a person has high perceived behavioral control, then they have an increased confidence that they are capable of performing the specific behavior successfully.

As show in the figure 1, a risk communicatoin approach will be used to deliever the hazard of smoking to the health and unborn baby to participants to increase the attitude towards quitting and subjective norm. Combining with the increasing of the two factors, the participants' intention to quit will enhance, and finally promt the participants to quit smoking.

Figure 1. The theoretical framework of smoking cessation based on the Heath Belief Model.

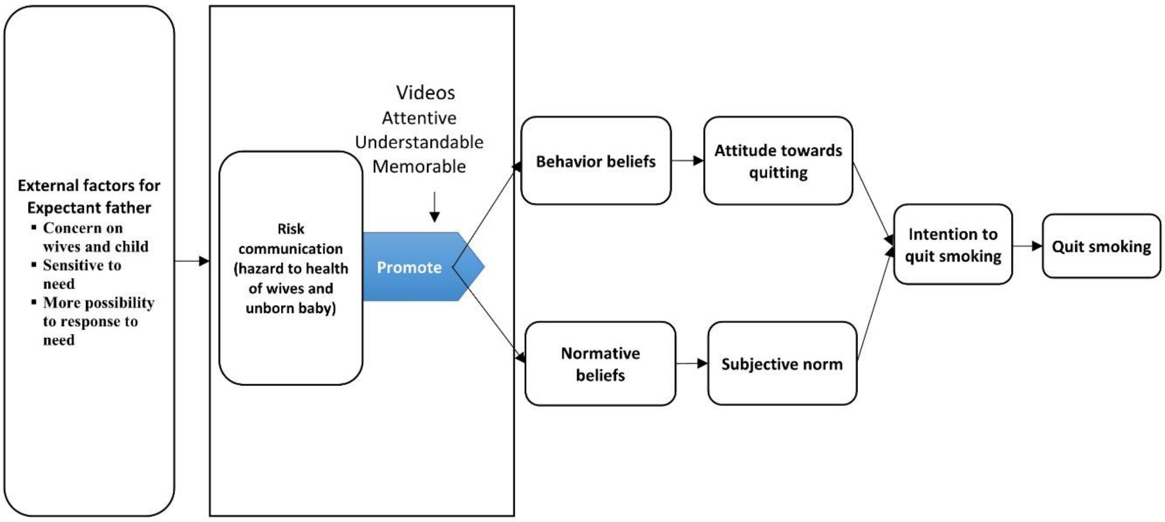

S. 3. Methodology

S. 3.1. Phase 1: Video developing

S. 3.1.1. Study Design

Before the intervention is implemented, a set of videos delivering the hazard of smoking will be developed. In this phase, four 1-minutes video will be produced that presented the health effects of smoking, especially emphasizing the importance of smoking cessation on the fetal and pregnancy. These videos will be drafted and then framed by the research staff.

S. 3.1.2. Evaluation

To holding subjects' interest, both static and dynamic pictures as well as short video clips will be used. The visuals will match and synchronize with the content of the auditory presentation to create coherent loss and gain. Auditory and visual materials will be produced professionally to optimize their quality. Finally, these videos will be evaluated by 3 healthcare professionals in this field before sending to the participants by smartphone stage by stage [57].

S. 3.2. Phase 2: Pilot study

S. 3.2.1. Study Design

Before the formal intervention is implemented, a pilot study will be applied to test the feasibility and acceptability of the video-led intervention.

S. 3.2.2. Sampling

Respectively 10 male smokers in Shenzhen who met the inclusion criteria for the study will

be invited to participate in this study. The inclusion criteria are: the participants should be (1) male resident aged 18 or above; (2) smoking at least one cigarette per day averagely during the past 3 months; (3) get married and partner has been pregnant; (4) able to communicate in Mandarin (including reading Chinese); and (5) have a smartphone for intervention and follow-up. Smokers who meet the above criteria but are currently involved in other smoking cessation programs and/or mentally or physically unfit for communication will be excluded.

S. 3.2.3 Measurement

S. 3.2.3.1. Demographics information sheet

The participants will be collected by using a self-designed demographics information questionnaire. The content of the demographics information sheets will include the age, gender, occupation, family income, education level, marriage status, the situation of child, the intend of pregnancy, patterner of smoking status, medical history, and alcohol using.

S. 3.2.3.2. Smoking characteristics

The baseline of smoking characteristics of the participants will be collected through a simplyfied questionnaire set which includes levels of daily cigarette consumption, history of past quit attempts.

S. 3.2.3.3. Structured interview

A ten minutes structured intervention aims to obtain the feasibility and acceptability of these videos will be applied to these participants. The interview will follow an outline about the evaluation and understanding for the vedios. The outline of the interview has been list in the table 1.

Table 1. Outline of structured interview for the feasibility and acceptability of video intervention.

1. Can you revieve the videos successfully or not? Please indicate the problems you faced during the video receiving.
2. Can you watch the videos smoothly or not? Please indicate which part or the possible reason that disturb you to watch the videos.
3. Can you understand the content of these videos or not? If not, please indicate it
4. Did you find any mistake in the video? Pealse indicate it/them.
5. Can you keep in watching the videos? Why or why not?
6. Do you think these videos are attractive or boring? Why?
7. Do you feel the image and of the videos are clear and visual confortable? If not, please indicate the part that you think is not clear and makes you unconforatble.
8. Does the sound in the videos is clear? If not, please indicate it.
9. Do you think these video are helpful for you? Please indicate which aspects you think is it

helpful.

10. Do you think the contents in the videos are acceptable? Why or why not?
11. Do you think the videos is helpful to quit smoking for you? why or why not?
12. Is there any part you think need to be deleted? Please indicate it.
13. Is there anything that you think can be added in these video?

S. 3.2.4. Data collection procedures

A convenient sampling method will be used in the pilot study. These eligible subjects will be invited to participate in the study after telling the purpose of this study. They will be given the option of participating or refusing and will be told that their participation is voluntary without prejudice. Written consent will be obtained from those subjects accepting to receive the interventions.

The subjects' demographic information, smoking characteristics will assessed by the research helpers. Then the ordered video will be sent to the participants through the smart-phone. After watching the video, the participants will be invited to attend a ten minutes invterview, the interview will be guided by research student and tape recorded.

S. 3.2.5. Data analysis

The feedback comments will be transcribe into the computer and summarized. Then the videos will be modified based on the result of pilot study.

S. 3.3. Phase 3: major study

S. 3.3.1. Study design

A parallel three-armed cluster randomized controlled trial will be used in this study to explore the efficient of a video-based intervention to the smoking cessation for male smokers preparing for pregnancy.

S. 3.3.2. Sampling

Male smokers, who met the inclusion criteria for the study in Shenzhen will be invited to participate. The inclusion criteria are: the participants should be (1) male resident aged 18 or above; (2) smoking at least one cigarette per day averagely during the past 1 months (exhaled CO verified); (3) get married and partner has been pregnant (no deformity); (4) able to communicate in Mandarin (including reading Chinese); and (5) have a smartphone for intervention and follow-up. Smokers who meet the above criteria but are currently involved in other smoking cessation programs or the pilot study and/or mentally or physically unfit for communication will be excluded.

Based on previous study conducted in Mainland China, the 6-month quit rate for the control group was approximately 14.8%[60, 61]. According to previous RCT of text message intervention, the rate ratio of quit rate for the intervention and control group was 2.18 (intervention group: 10.7%, control

group: 4.9 %)[62]. To detect a significant difference of quit rate between intervention and control groups with a power of 80 and 5 % significance level, 264 subjects per group will be needed. Assuming an retention rate of 80% at the 6-month follow-up, the total sample size taking into account in the recruitment session and attrition is 990.

S. 3.3.3. Measurements

S. 3.3.3.1. Demographics information sheet

The participants will be collected by using a self-designed demographics information questionnaire which is the same with the one used in the pilot study. The content of the demographics information sheets will include the age, gender, occupation, family income, education level, the number of child, partners' smoking status, medical history, and alcohol using.

S. 3.3.3.2. Smoking characteristics

The baseline of smoking characteristics will be collected through a structured questionnaire set. The content of the structured questionnaire set will include the, levels of daily cigarette consumption, history of past quit attempts, nicotine dependency assessed by the Fagerstrom Test of Nicotine Dependence.

S.3.3.3.3. The Fagerstrom Test of Nicotine Dependence (FTND)

The FTND is a standard instrument for assessing the intensity of physical addiction to nicotine, developed by Karl-Olov Fagerström and modified by Todd Heatherton [63, 64].The test was designed to provide an ordinal measure of nicotine dependence related to cigarette smoking. It contains six items that evaluate the quantity of cigarette consumption, the compulsion to use, and dependence. In scoring the Fagerstrom Test for Nicotine Dependence, yes/no items are scored from 0 to 1 and multiple-choice items are scored from 0 to 3. The items are summed to yield a total score of 0-10. The higher the total Fagerstrom score, the more intense is the patient's physical dependence on nicotine.

S.3.3.3.4. Smoking Self-efficacy Questionnaire (SEQ-12)

Self-efficacy of participants against tobacco will be assessed by using by the SEQ-12. The SEQ-12 is categorized into two subscales, namely internal stimuli (6 items) and external stimuli (6 items), with total possible scores ranging from 6 to 30 for both internal stimuli and external stimuli. Higher scores of the SEQ-12 on both subscales indicate greater self-efficacy to refrain from smoking.

The psychometric properties of this scale have been empirically examined with the Cronbach's alpha coefficients of 0.95 and 0.94 for internal stimuli and external stimuli, respectively, indicating excellent internal consistency. The intraclass correlation coefficients were 0.95 and 0.93 for internal stimuli and external stimuli, respectively, demonstrating excellent test-retest reliability[2].

S. 3.3.3.5. the Short Form 12 (SF-12) Health Survey

The short form 12 (SF-12) health survey has been extensively studied and used as a valid measure of health-related quality of life in a variety of population groups, no systematic studies have described the reliability of the measure in patients with behavioral conditions or serious mental illness (SMI).

S.3.3.1. Interventions

S. 3.3.4.1. Smoking hazard videos+brief advice+Pamphlet

The videos which presented the health effects of smoking, especially emphasizing the importance of smoking cessation on the fetal and pregnancy, will be sent through the smartphone to the participants in the intervention group 1. Also the participants will receive a brief quit advice and pamphlet material related to the smoking cessation at the baseline.

S. 3.3.4.2. Smoking hazard text-messageing+brief advice +Pamphlet

Text message with the same context with the video will be sent to the participants in the intervention group 2. Also the participants will receive a brief quit advice and pamphlet material related to the smoking cessation at the baseline.

S. 3.3.4.3. Brief advice+Pamphlet

Participants in the control group will be given a very brief, minimal and general smoking cessation advice during the first assessment. A pamphlet showed the information related to the smoking cessation will be allocated to them at the same time.

S. 3.3.4.5. Follow-up

All participants will be followed up at one week, one-month, three-month and six-month point by telephone. And the 6-month point is also the end point of the follow up.

S. 3.3.5. Validation of quitting

The level of exhaled carbon monoxide (CO) level will be used as the biomedical marker for assessing the smoking cessation [65].

S. 3.3.6. Outcome assessments

S. 3.3.6.1. Primary outcome

The primary outcomes are self-reported 7-days point prevalence quit rate at 6-month. Subjects reporting not smoking in the past 7-days at 6-month will be regarded as abstinence from smoking.

S. 3.3.6.2. Secondary outcome

1. Biomedical validated smoking abstinence: smoking abstinence will be defined as exhaled carbon monoxide (CO) level < 4ppm
2. Smoking reduction: cigarette consumption reduced by at least 50% compared with the

baseline at 6-month.

3. Level of readiness to quit smoking at 6 month

S. 3.3.7. Data collection procedures

Male smokers will be recruited in the Obstetrics and Gynecology clinics at tertiary public hospitals in China when the males accompany with their parterners to have a body check. Invitation letter will be sent to the public hospitals to participate in this study. After obtain the permission of these hospitals. These eligible people were invited to participate in the study after telling the purpose of this study. They will be given the option of participating or refusing and will be told that their participation is voluntary without prejudice. Written consent will be obtained from those subjects accepting to receive the interventions.

After the consent is obtained, the participant will be coded. By using a computer random selection procedure from the code, the participants will be randomly allocated into three arms, which are intervention group 1, intervention group 2 and control group. The subjects' demographic information, smoking characteristics, self-efficacy level, and exhaled carbon monoxide (CO) level at the baseline will assessed by the research nurses. The biochemical validation will be done by a staff member who has not delivered the intervention and conducted the telephone counselling follow-up. Yet all subjects will not be blinded, because they will receive the behavioral intervention.

In the intervention group, the smokers will receive an intervention including video, brief advice, and pamphlet. In the intervention group 2, the subjects will text message, brief advice and pamphlet. For the participants in the control group, they will only receive a brief advice and pamphlet materials at the baseline. The detail procedure of data collection is presented in the figure 2 below.

S. 3.4. Data analysis

The IBM SPSS Statistics (SPSS: Version 20.0; SPSS Inc., Chicago, IL, USA) for windows will be used for data analysis. A p-value <0.05 was considered as statistically significant. Descriptive statistics will be used to calculate the frequency and percentage (categorical data) or the mean and standard deviations (continuous data) of different demographic and social-economic characteristics. The intention-to-treat (ITT) principle will be used for outcome comparison between groups. Information missed at follow-up will be considered as non-quitters, non-reducers or do not use the video if no records about watching full video set. Multiple imputation will be used to fill the missing value of the demographic information at baseline. One-way ANOVA test or Mann- Whitney test and chi-square test will be used to compare the differences of nicotine dependency, stage of readiness to quit, and self-efficacy among three groups between baseline and 6-month follow-up for those who continued smoking. Logistic multiple regression will be conducted to examine the association between the intervention and the outcomes at 6-month follow-up. Subgroup analysis will be

performed by the first time to be father, level of nicotine dependence, and readiness to quit at baseline.

S. 3.5. Ethical Considerations

Ethics approval of the study will obtain from Institutional Review Board (IRB) of the University of Hong Kong. The interventions have no physical impact on the subjects. If the participants have any psychological uncomfortable, like depression or anxiety, professional counselling will be provided to help them to decrease these reactions. And they can drop out this study at any time.

S.4. Significance, Expected result and Implementation

The results of the study can verify the efficient of the video based smoking cessation intervention for the male smokers preparing to pregnancy, and provide essential complementary information on the baseline of self-efficacy about the smoking cessation for male smokers. Besides, result of this study will provide the evidence that a smartphone video is an efficient media which can improve the feasibility of smoking cessation intervention.

The findings from this study can provide information to the health professionals. Then they can adjust the implementation and policy, and further develop appropriate intervention to take fully advantage of family members to help the smokers to quit smoking more efficiently.

Figure 2. The procedure of data collection

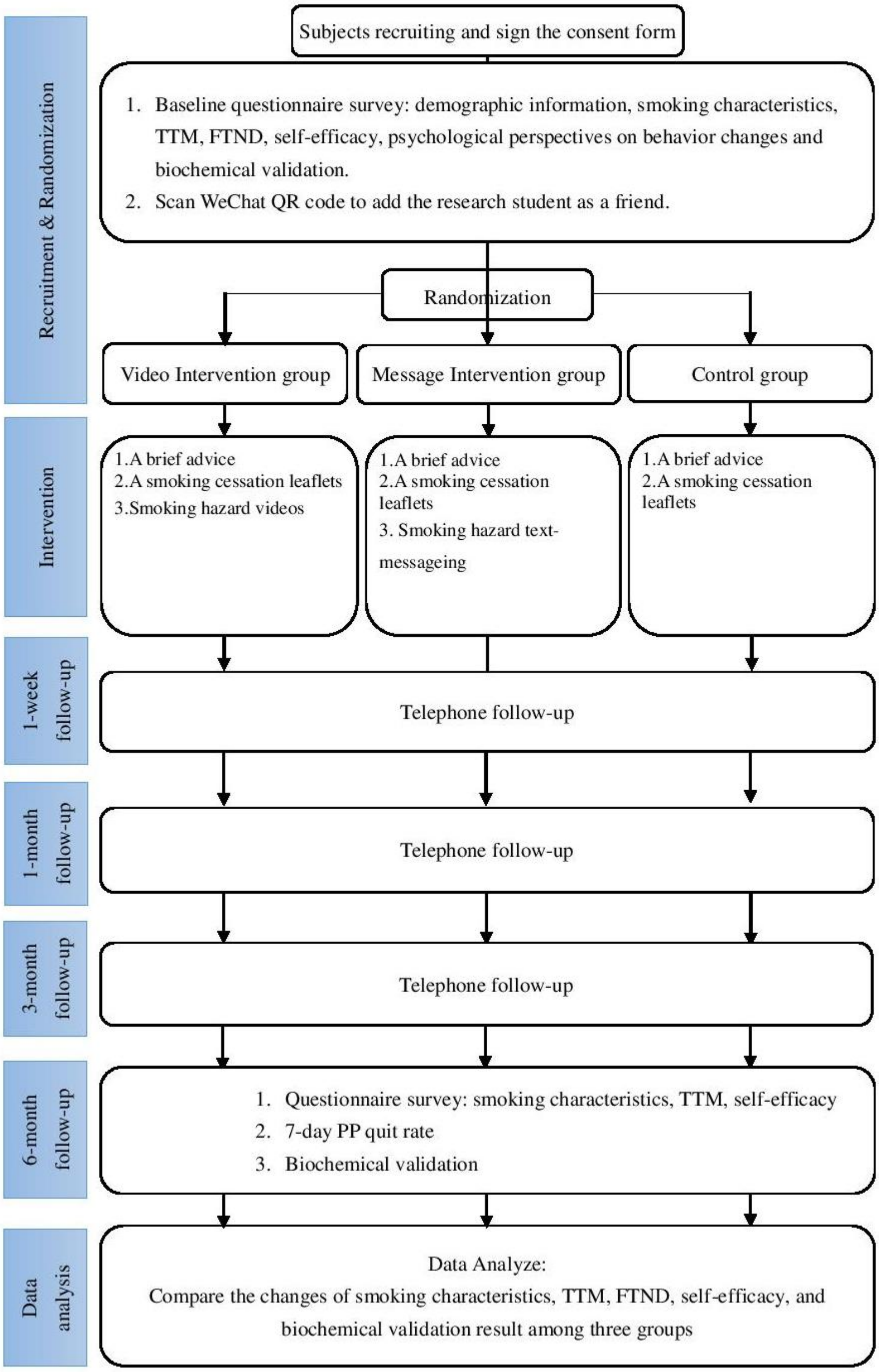

S.5. Chronological outline of research plan

Time(month)	01 (03/2017)	02	03	04	05	06	07	08	09	10	11	12	13	14	15	16	17	18	19	20	21	22	afterwards
Cross-sectional study<br>Investigation																							
Data analysis																							
Video making																							
Pilot study<br>Recruitment																							
Intervention																							
Follow-up																							
Data analysis																							
Study design reversion																							
Major study<br>Recruitment																							
Apply intervention																							
Follow up																							
Data analysis																							
Outcome report																							

References:

1. Giovino, G.A., et al., *Tobacco use in 3 billion individuals from 16 countries: an analysis of nationally representative cross-sectional household surveys.* The Lancet, 2012. **380**(9842): p. 668-679.
2. Jean-Francois Etter, et al., *Development and validation of a scale measuring self-efficacy of current and former smokers.* Addiction, 2000. **95**(6): p. 901-913.
3. Jha, P. and R. Peto, *Global effects of smoking, of quitting, and of taxing tobacco.* N Engl J Med, 2014. **370**(1): p. 60-8.
4. Zhang, J., J.X. Ou, and C.X. Bai, *Tobacco smoking in China: prevalence, disease burden, challenges and future strategies.* Respirology, 2011. **16**(8): p. 1165-72.
5. Finucane, M.M., et al., *National, regional, and global trends in body-mass index since 1980: systematic analysis of health examination surveys and epidemiological studies with 960 country-years and 9·1 million participants.* The Lancet, 2011. **377**(9765): p. 557-567.
6. Feigin, V., et al., *Smoking and elevated blood pressure are the most important risk factors for subarachnoid hemorrhage in the Asia-Pacific region: an overview of 26 cohorts involving 306,620 participants.* Stroke, 2005. **36**(7): p. 1360-5.
7. Fiorini, T., et al., *Is there a positive effect of smoking cessation on periodontal health? A systematic review.* J Periodontol, 2014. **85**(1): p. 83-91.
8. Wang, J.B., et al., *Estimation of cancer incidence and mortality attributable to smoking in China.* Cancer Causes Control, 2010. **21**(6): p. 959-65.
9. Islami, F., L.A. Torre, and A. Jemal, *Global trends of lung cancer mortality and smoking prevalence.* Transl Lung Cancer Res, 2015. **4**(4): p. 327-38.
10. A.J. Sascoa and K.S. Secretana, *Tobacco smoking and cancer: a brief review of recent epidemiological evidence.* Lung Cancer, 2004. **45**(2): p. S3-S9.
11. Cao, S., et al., *Association of quantity and duration of smoking with erectile dysfunction: a dose-response meta-analysis.* J Sex Med, 2014. **11**(10): p. 2376-84.
12. Cao, S., et al., *Smoking and risk of erectile dysfunction: systematic review of observational studies with meta-analysis.* PLoS One, 2013. **8**(4): p. e60443.
13. Kevin T. Mcvary, Serge Carrier, and H. Wessells, *Smoking and Erectile Dysfunction: Evidence Based Analysis.* The Journal of Urology. **166**(5): p. 1624-1632.
14. Zhi Hong Zhang, et al., *Decline of semen quality and increase of leukocytes with cigarette smoking in infertile men.* Iran J Reprod Med, 2013. **11**(7): p. 589-596.
15. Asare-Anane, H., et al., *Tobacco smoking is associated with decreased semen quality.* Reprod Health, 2016. **13**(1): p. 90.
16. Goodchild, M., & Zheng, R. Tobacco control and Healthy China 2030. Tob Control, 2018. tobaccocontrol-2018-054372. doi:10.1136/tobaccocontrol-2018-054372
17. Taha, E.A., et al., *Effect of smoking on sperm vitality, DNA integrity, seminal oxidative stress, zinc in fertile men.* Urology, 2012. **80**(4): p. 822-5.
18. Fang, S.C., et al., *Validity of Self-Reported Tobacco Smoke Exposure among Non-Smoking Adult Public Housing Residents.* PLoS One, 2016. **11**(5): p. e0155024.
19. Matt, G.E., et al., *Thirdhand smoke and exposure in California hotels: non-smoking rooms fail to protect non-smoking hotel guests from tobacco smoke exposure.* Tob Control, 2014. **23**(3): p. 264-72.

20. Nelson-Piercy, C., *Pre-pregnancy counselling.* Current Obstetrics & Gynaecology, 2003. **13**(5): p. 273-280.
21. Heather Wipfli, et al., *Secondhand Smoke Exposure Among Women and Children: Evidence From 31 Countries.* American Journal of Public Health, 2008. **98**(4): p. 672-680.
22. Hyland, A., et al., *Associations of lifetime active and passive smoking with spontaneous abortion, stillbirth and tubal ectopic pregnancy: a cross-sectional analysis of historical data from the Women's Health Initiative.* Tob Control, 2015. **24**(4): p. 328-35.
23. Iniguez, C., et al., *Active and passive smoking during pregnancy and ultrasound measures of fetal growth in a cohort of pregnant women.* J Epidemiol Community Health, 2012. **66**(6): p. 563-70.
24. Qiu, J., et al., *Passive smoking and preterm birth in urban China.* Am J Epidemiol, 2014. **180**(1): p. 94-102.
25. Leonardi-Bee, J., et al., *Environmental tobacco smoke and fetal health: systematic review and meta-analysis.* Arch Dis Child Fetal Neonatal Ed, 2008. **93**(5): p. F351-61.
26. Hernandez-Martinez, C., et al., *A longitudinal study on the effects of maternal smoking and secondhand smoke exposure during pregnancy on neonatal neurobehavior.* Early Hum Dev, 2012. **88**(6): p. 403-8.
27. Lee, B.E., et al., *Secondhand smoke exposure during pregnancy and infantile neurodevelopment.* Environ Res, 2011. **111**(4): p. 539-44.
28. Richard Doll, et al., *Mortality in relation to smokingL 50 years' observations on ,ale British doctors.* BMJ: British Medical Journal, 2004. **328**(7455): p. 1519-1528.
29. Sakata, R., et al., *Impact of smoking on mortality and life expectancy in Japanese smokers: a prospective cohort study.* BMJ, 2012. **345**: p. e7093.
30. Pirie, K., et al., *The 21st century hazards of smoking and benefits of stopping: a prospective study of one million women in the UK.* The Lancet, 2013. **381**(9861): p. 133-141.
31. Yang, J., et al. "Health knowledge and perception of risks among Chinese smokers and non-smokers: findings from the Wave 1 ITC China Survey." *Tob Control*. 2010. 19.Suppl 2: i18-i23.
32. Yang, L., et al., *Exposure to secondhand smoke and associated factors among non-smoking pregnant women with smoking husbands in Sichuan province, China.* Acta Obstet Gynecol Scand, 2010. **89**(4): p. 549-57.
33. Kenkel, D., D.R. Lillard, and F. Liu, *An analysis of life-course smoking behavior in China.* Health Econ, 2009. **18 Suppl 2**: p. S147-56.
34. Yang GH , et al., *Smoking and passive smoking in Chinese, 2002.* Zhonghua liu xing bing xue za zhi= Zhonghua liuxingbingxue zazhi, 2005. **26**(2): p. 77-83.
35. Gong-Huan Yang, et al., *Findings from 2010 global adult tobacco survey: Implementation of MPOWER policy in China.* Biomedical and Environmental Sciences, 2010. **23**(6): p. 422-429.
36. Ng, M., et al., *Smoking prevalence and cigarette consumption in 187 countries, 1980-2012.* JAMA, 2014. **311**(2): p. 183-92.
37. Veeranki, S.P., et al., *Secondhand smoke exposure among never-smoking youth in 168 countries.* J Adolesc Health, 2015. **56**(2): p. 167-73.
38. Ahmed, M., et al., *Secondhand smoke exposure among nonsmoker adult females in rural households of Aligarh.* International Journal of Medical Science and Public Health, 2015: p. 1.
39. King, B.A., et al., *A cross-country comparison of secondhand smoke exposure among adults:*

findings from the Global Adult Tobacco Survey (GATS). Tob Control, 2013. **22**(4): p. e5.
40. Zhang, L., et al., *Exposure to secondhand tobacco smoke and interventions among pregnant women in China: a systematic review.* Prev Chronic Dis, 2015. **12**: p. E35.
41. Tingting Yao, et al., *Secondhand smoke exposure at home in rural China.* Cancer Causes Control, 2012. **23**(1): p. 109-115.
42. Gruder, C.L., et al., *Tobacco smoking, quitting, and relapsing among adult males in Mainland China: the China Seven Cities Study.* Nicotine Tob Res, 2013. **15**(1): p. 223-30.
43. Jiang, Y., et al., *Quitting smoking in China: findings from the ITC China Survey.* Tob Control, 2010. **19 Suppl 2**: p. i12-7.
44. Yan Yang, et al., *Awareness of Tobacco-Related Health Hazards among Adults in China.* Biomedical and environment sicence, 2010. **23**(6): p. 437-444.
45. Kim, S.S., et al., *A systematic review of smoking cessation intervention studies in China.* Nicotine Tob Res, 2012. **14**(8): p. 891-9.
46. Bandura, A., *Self-efficacy: Toward a unifying theory of behavioral change.* Psychological review, 1977. **84**(2): p. 191-215.
47. Condiotte, M.M., *Self-efficay and relapse in smoking cessation programs.* Journal of Consulting and Clinical Psychology, 1981. **49**(5): p. 648-658.
48. Suzy B. Gulliver, et al., *An investigation of self-efficacy, partner support and daily stresses as predictors of relapse to smoking in self-quitters.* Addiction, 1995. **90**(2): p. 767-772.
49. Gao, Y., et al., *Parental migration, self-efficacy and cigarette smoking among rural adolescents in south China.* PLoS One, 2013. **8**(3): p. e57569.
50. Bandura, A. and E.A. Locke, *Negative self-efficacy and goal effects revisited.* Journal of Applied Psychology, 2003. **88**(1): p. 87-99.
51. Hiscock, R., et al., *Socioeconomic status and smoking: a review.* Ann N Y Acad Sci, 2012. **1248**: p. 107-23.
52. Yong, H.H., et al., *Urban Chinese smokers from lower socioeconomic backgrounds face more barriers to quitting: results from the international tobacco control-China survey.* Nicotine Tob Res, 2013. **15**(6): p. 1044-51.
53. Westmaas, J.L., J. Bontemps-Jones, and J.E. Bauer, *Social support in smoking cessation: reconciling theory and evidence.* Nicotine Tob Res, 2010. **12**(7): p. 695-707.
54. Zhang, J.Y., et al., *The social context of smoking cessation in China: an exploratory interview study.* Tob Control, 2012. **21**(1): p. 57-8.
55. Wagner, J., M. Burg, and B. Sirois, *Social support and the transtheoretical model: Relationship of social support to smoking cessation stage, decisional balance, process use, and temptation.* Addict Behav, 2004. **29**(5): p. 1039-43.
56. Mao, A., K. Bristow, and J. Robinson, *Caught in a dilemma: why do non-smoking women in China support the smoking behaviors of men in their families?* Health Educ Res, 2013. **28**(1): p. 153-64.
57. Yang, T., et al., *Attitudes to smoking cessation and triggers to relapse among Chinese male smokers.* BMC Public Health, 2006. **6**: p. 65.
58. Schneider, T.R., et al., *Visual and Auditory Message Framing Effects on Tobacco Smoking.* Journal of Applied Social Psychology, 2001. **31**(4): p. 667-682.
59. Ajzen, I., *The Theory of Planned Behavior.* Organizational Behavior and Human Decision Progresses, 1991. **50**: p. 179-211.

60. Li, W.H., et al., *Helping cancer patients quit smoking by increasing their risk perception: a study protocol of a cluster randomized controlled trial.* BMC Cancer, 2015. **15**: p. 490.
61. Qiang Li, Jason Hsia, and Gonghuan Yang, *Prevalence of Smoking in China in 2010.* New England Journal of Medicine, 2010. **364**(25): p. 2469-2470.
62. Wu, L., et al., *The effect of a very brief smoking-reduction intervention in smokers who have no intention to quit: study protocol for a randomized controlled trial.* BMC Public Health, 2015. **15**: p. 418.
63. Wu, L., et al., *Effectiveness of additional follow-up telephone counseling in a smoking cessation clinic in Beijing and predictors of quitting among Chinese male smokers.* BMC Public Health, 2016. **16**: p. 63.
64. Pomerleau, C.S., M.J. Majchrzak, and O.F. Pomerleau, *Nicotine dependence and the Fagerström Tolerance Questionnaire: A brief review.* Journal of Substance Abuse, 1989.
65. Benowitz, N.L., et al., *Biochemical verification of tobacco use and cessation.* Nicotine Tob Res, 2002. **4**(2): p. 149-59.
