# Supplementary material for: Effectiveness of a video-based smoking cessation intervention focusing on maternal and child health in promoting quitting among expectant fathers in China: A randomized controlled trial
Source: PLoS Med. 2020 Sep 29;17(9):e1003355. doi: 10.1371/journal.pmed.1003355 (PMC7523971; doi:10.1371/journal.pmed.1003355)
Supplement: S2 Text — (DOCX) [file pmed.1003355.s003.docx]

## S2 Text. Translated content of the interventional videos and text

The translated videos were available online via the link below:

**Link:**

<https://lecturecapture.hku.hk/Panopto/Pages/Sessions/List.aspx?folderID=e7bbd983-296d-42c8-b1bf-ab1b0041c7bb>

**Translated content of the interventional videos and text**

Video 1/ Text 1

Cigarettes are among the main killers of people worldwide. One of every two smokers loses their life prematurely because of smoking. Smoking can cause cough, coronary heart disease, stroke, liver disease, stomach ulcers, and other diseases. In addition, smoking can induce lung cancer, laryngeal cancer, oral cancer, colon cancer, and other cancers. Smoking can aggravate arthritis and back pain as well as cause impotence and infertility owing to sperm deformity and reduced activity. Do not wait until these problems appear. To remain healthy for yourself and your children for many years to come, please quit smoking now.

Video 2/Text 2

When you smoke near a pregnant woman, the fetus in her womb is smoking with you. Pregnant women who actively or passively smoke during pregnancy have three to four times the likelihood of stillbirth. When pregnant women are exposed to secondhand smoke for a long time, harmful substances in the smoke enter the maternal blood, which decreases the blood oxygen content. The fetus becomes prone to growth retardation owing to a lack of oxygen in the uterus, and the infant’s birth weight will be lower than normal. A fetus with a history of exposure to smoking has a greatly increased risk of deformities such as cleft lip and palate, hernia, and limb deformity, as well as cardiovascular disease and respiratory distress, among other health problems. Please listen to your unborn child's voice. So that you child can have a healthy life, please quit smoking.

Video 3/Text 3

When you smoke around children, they are exposed to a large number of chemicals that destroy their health. The smoke and ashes from cigarettes contain toxic substances such as hydrogen cyanide and carbon monoxide, which can cause serious health problems in children, such as ear canal infections, lifelong asthma, and pneumonia. Smoking exposure is also associated with low birth weight, which doubles the risk of sudden infant death syndrome. Tobacco is destroying your life and that of your baby. Quit smoking now!

Video 4/Text 4

The dangers of smoking for your family cannot be ignored. Secondhand smoke reduces the environmental quality in your home and also increases the risks for your pregnant partner. When you smoke, nicotine particles stick to clothing and furniture, which are difficult to clean. Adsorbed nicotine releases harmful substances for a long time, which is known as thirdhand smoke. This constantly threatens the health of pregnant women and children. Smoking outside the home does not prevent the harm caused by thirdhand smoke; the only solution is to quit smoking. Do not wait until your baby is born. Create a clean home for your child and stop smoking now.
