# Supplementary material for: Effectiveness of a video-based smoking cessation intervention focusing on maternal and child health in promoting quitting among expectant fathers in China: A randomized controlled trial
Source: PLoS Med. 2020 Sep 29;17(9):e1003355. doi: 10.1371/journal.pmed.1003355 (PMC7523971; doi:10.1371/journal.pmed.1003355)
Supplement: S4 Text — (PDF) [file pmed.1003355.s005.pdf]

S4 Text. Follow-up questionnaires

1 week

Code： ________________ Date： _________________Name:____________Contact number: _____________

Contact history:

**Call attempt**	**1**	**2**	**3**	**4**	**5**	**6**	**7**
**Caller**							
**Date/time**							
**Status**							

Part One：Smoking status

A1. Have you smoked in the past 7 days? 1.☐ No, I did not smoke 0.☐ Yes

A1-0-1 How long have you sustained? ______days

A1-1-1.In the past 7 days, How many days do you smoke, Average _____days
A1-1-2.In the past 7 days, How many cigarettes do you smoke on working days? Average ___Cigarettes /day
How many cigarettes do you smoke on weekend? Average ___Cigarettes /day

A2. Do you use e-cigarettes in the past 7 days? 1.☐ Yes 0.☐ No (Go to A6）

A3. Does the e-cigarette you used contain nicotine ingredients?
1.☐ Yes 0.☐ No 9. ☐ Don't know

A4. Do you use heated tobacco products in the past 7 days? 1.☐ Yes 0.☐ No

A5. Do you use any other tobacco products in the past 7 days except for traditional cigarettes, electronic cigarettes or heated cigarettes?
0.☐ No (Go to A9） 1.☐ Yes, It's______（Please fill the category of the tobacco products）

A6. How many such tobacco products have you used in the past past 7 days?
A total of _____ times, a total of ______units (please fill in the specific number on the line)\

A7. How soon after waking do you smoke your first cigarete?
3.☐ Within 5 mins 2.☐ 6-30 mins 1.☐ 31-60 mins 0.☐ 60 mins later

A8. Do you find it difficult to refrain from smoking places where it is forbidden? E.g. Church, Library, etc.
1. ☐ Yes 0. ☐ No

A9. Which cigarette would you hate to give up? 1. ☐ The First in the morning 0. ☐ Any other

A10. How many cigarette a day do you smoke?
0. ☐ 10 or less 1. ☐ 11-20 2. ☐ 21-30 3. ☐ 31 or more

A11. Do you smoke more frequently in the morning? 1. ☐ Yes 0. ☐ No

A12. Do you smoke even if you are sick in most of the day? 1. ☐ Yes 0. ☐ No

0To be completed by researcher: Total score: _______
Nicotine Dependency: 1.☐ Mild (0–3) 2.☐ Moderate (4–5) 3.☐ Severe (6 – 10)

Part Two: Quit history

B1. Since last meet, how many times have you attempted to quit smoking for more than 24 hours in the past year?
0. ☐ No attempt (Go to D1) 1. ☐ Yes, I attempted________times (Please fill in the specific number on the line)

B2. Since last meet, how long have you sustained to no smoke?________days (Please fill in the specific number on the line)

C1. Do you plan to quit smoking now? 1. ☐ Yes 0. ☐ No(Pre-contemplation) (Go to D1)

C2. When do you plan to quit smoking? (Single choice)

0. ☐ I have no smoking now (action)
1. ☐ I will quit smoking within 7 days (preparation/contemplation)
2. ☐ I will quit smoking within 1 month (preparation/contemplation)
3. ☐ I will quit smoking within 6 months (contemplation)
4. ☐ I will quit smoking after 6 months (pre-contemplation)
5. ☐ Have not made a decision yet (pre-contemplation)

The exact time
i)___ days after; or
ii)___ months after.

C3. 1. ☐ Decided smoking cessation date ____________________ 0. ☐ Undecided smoking cessation date

0 Stage of readiness to quit smoking:
☐ Action ☐ Preparation# ☐ Contemplation# ☐ Pre-contemplation
Preparation: At least one 24-hour quit attempt in the past year; **Contemplation:** No quit attempt

Part Three: The use of smoking cessation services

D1. Since last meet, have you used any other smoking cessation service in the past year?
1. ☐ Yes 0. ☐ No (Go to part seven)

D2. What smoking cessation service you have used? (Multiple choices)

	No	Yes	(times used)
1. ☐ Telephone counselling	☐	☐	(_________)
2. ☐ Nicotine replacement treatment	☐	☐	(_________)
3. ☐ Face to Face counselling	☐	☐	(_________)
4. ☐ Group discussion	☐	☐	(_________)
5. ☐ Medication treatment	☐	☐	(_________)
6. ☐ Acupuncture treatment	☐	☐	(_________)
7. ☐ Others：_____________________ (Please mark)	☐	☐	(_________)

Thank you for your cooperation! This is the end of the questionnaire

1 周随访问卷

编码：____________ 日期：______________姓名：_________联系电话：_________

联络记录：

尝试次数	1	2	3	4	5	6	7
填写人							
日期/时间							
状态							

第一部分：吸烟情况

A1. 您最近7天内有没有吸烟？ 0 ☐ 没有，一口都没有 1 ☐ 有

A1-0-1 您有多长时间一点都没有吸烟？________天

A1-1-1 最近7天内，您大概有几天有吸烟？_____ 天

A1-1-2 过去的30天内，工作日通常一天吸_____ 支；周末吸烟，通常一天吸_____ 支

A2. 您最近7天内有没有使用电子烟？1.☐ 有 0.☐ 没有(跳到A6）

A3. 您使用的电子烟含不含尼古丁成分？1.☐ 有 0.☐ 没有 9. ☐ 不知道

A4. 您最近7天内有没有使用加热烟？1.☐ 有 0.☐ 没有

A5. 除了传统烟、电子烟或者加热烟，您最近7天内还有没有使用其他烟草产品？
0.☐ 没有 (跳到D部分） 1.☐ 有，是__________(请填入烟草产品)

A6. 你最近7天内有使用多少这种烟草产品？共_____次，共______个/支（请在横线上填入具体数字）

A7. 您早晨醒来后多久吸第一支煙？
3.☐ 5 分钟 2.☐ 6-30 分钟内 1.☐ 31-60 分钟内 0.☐ 60 分钟后

A8. 当您身处非吸烟区内，会不会感到难受而忍不住吸烟？ 1. ☐ 会 0. ☐ 不会

A9. 您觉得哪一支烟最难放弃？ 1. ☐早上第一支 0. ☐其它

A10. 您每天抽多少支烟？
0. ☐ 10支或以下 1. ☐ 11-20支 2. ☐ 21-30支 3. ☐ 31支或更多

A11. 当您生病几乎整天卧病在床时还会吸烟吗？ 1. ☐ 会 0. ☐ 不会

A12. 在起床后数小时内，您吸烟次数是否较在其他时间频繁？ 1. ☐ 会 0. ☐ 不会

0To be completed by researcher: Total score: _______
Nicotine Dependency: 1.☐ Mild (0–3) 2.☐ Moderate (4–5) 3.☐ Severe (6 – 10)

第二部分：戒烟史部分：戒烟情况

B1. 自从上次见面之后，有没有认真试过连续24小时以上不吸烟？1 ☐ 有试过 0 ☐ 没有试过

B2. 自从上次见面之后，你最长有多久没有吸烟？ ________天

C1. 您现在有没有打算戒烟？1. ☐ 有 0. ☐ 没有(Pre-contemplation) (跳去 D1)

C2. 您打算从什么时候开始戒烟？(单选)

0. ☐ 我现在已经没有吸烟了 (action) → 确切时间： i)___ 日后 或 ii)___ 月后
1. ☐ 我会在近7天之内戒烟 (preparation/contemplation)
2. ☐ 我会在近1个月之内戒烟 (preparation/contemplation)
3. ☐ 我会在近6个月之内戒烟 (contemplation)
4. ☐ 我会在6个月之后戒烟 (pre-contemplation)
5. ☐ 还没做好决定 (pre-contemplation)

C3. 1. ☐ 已决定的戒烟日期____________________ 0. ☐ 未决定戒烟日期

0 Stage of readiness to quit smoking:

☐ Action ☐ Preparation# ☐ Contemplation# ☐ Pre-contemplation

Preparation: At least one 24-hour quit attempt in the past year; **Contemplation:** No quit attempt

第三部分：戒烟辅导服务使用情况

D1. 自从上次见面之后，你有没有用过任何其他戒烟辅导服务？

1. ☐ 有 0. ☐ 没有（问卷完）

D2. 您使用的是什么服务？（可多选）

	没有	有	（次数）
1. ☐ 电话戒烟辅导	☐	☐	（____）
2. ☐ 尼古丁替代治疗	☐	☐	（____）
3. ☐ 面对面辅导	☐	☐	（____）
4. ☐小组讨论戒烟辅导	☐	☐	（____）
5. ☐ 戒烟药物治疗	☐	☐	（____）
6. ☐ 针灸治疗	☐	☐	（____）
7. ☐ 其他：_____________________（请注明）	☐	☐	（____）

——————————多谢您的合作！问卷到此结束——————————

Follow-up Questionnaire: 1 month

Code：________________ Date：_________________Name:____________Contact number: _____________

Contact history:

**Call attempt**	**1**	**2**	**3**	**4**	**5**	**6**	**7**
**Caller**							
**Date/time**							
**Status**							

Part One：Smoking status

A1. Have you smoked in the past 7 days? 1.☐ No, I did not smoke 0.☐ Yes

A1-0-1 How long have you sustained? ______days

A1-1-1.In the past 30 days, How many days do you smoke, Average _____days

A1-1-2.In the past 30 days, How many cigarettes do you smoke on working days? Average ___Cigarettes /day

How many cigarettes do you smoke on weekend? Average ___Cigarettes /day

A2. Do you use e-cigarettes in the past 30 days? 1.☐ Yes 0.☐ No (Go to A6）

A3. Does the e-cigarette you used contain nicotine ingredients?

1.☐ Yes 0.☐ No 9. ☐ Don't know

A4. Do you use heated tobacco products in the past 30 days? 1.☐ Yes 0.☐ No

A5. Do you use any other tobacco products in the past 30 days except for traditional cigarettes, electronic cigarettes or heated cigarettes?

0.☐ No (Go to A9） 1.☐ Yes, It's______（Please fill the category of the tobacco products）

A6. How many such tobacco products have you used in the past 30 days?

A total of _____ times, a total of ______units (please fill in the specific number on the line)\

A7. How soon after waking do you smoke your first cigarete?

3.☐ Within 5 mins 2.☐ 6-30 mins 1.☐ 31-60 mins 0.☐ 60 mins later

A8. Do you find it difficult to refrain from smoking places where it is forbidden? E.g. Church, Library, etc.

1. ☐ Yes 0. ☐ No

A9. Which cigarette would you hate to give up? 1. ☐ The First in the morning 0. ☐ Any other

A10. How many cigarette a day do you smoke?

0. ☐ 10 or less 1. ☐ 11-20 2. ☐ 21-30 3. ☐ 31 or more

A11. Do you smoke more frequently in the morning? 1. ☐ Yes 0. ☐ No

A12. Do you smoke even if you are sick in most of the day? 1. ☐ Yes 0. ☐ No

0To be completed by researcher: Total score: _______

Nicotine Dependency: 1.☐ Mild (0–3) 2.☐ Moderate (4–5) 3.☐ Severe (6 – 10)

Part Two: Quit history

B1. Since last call, how many times have you attempted to quit smoking for more than 24 hours in the past year?

0. ☐ No attempt (Go to D1) 1. ☐ Yes, I attempted________times (Please fill in the specific number on the line)

B2. Since last call, how long have you sustained to no smoke?________days (Please fill in the specific number on the line)

C4. Do you plan to quit smoking now? 1. ☐ Yes 0. ☐ No(Pre-contemplation) (Go to D1)

C5. When do you plan to quit smoking? (Single choice)

0. ☐ I have no smoking now (action)
1. ☐ I will quit smoking within 7 days (preparation/contemplation)
2. ☐ I will quit smoking within 1 month (preparation/contemplation)
3. ☐ I will quit smoking within 6 months (contemplation)
4. ☐ I will quit smoking after 6 months (pre-contemplation)
5. ☐ Have not made a decision yet (pre-contemplation)

The exact time
i)___ days after; or
ii)___ months after.

C6. 1. ☐ Decided smoking cessation date ____________________ 0. ☐ Undecided smoking cessation date

0 Stage of readiness to quit smoking:
☐ Action ☐ Preparation# ☐ Contemplation# ☐ Pre-contemplation
Preparation: At least one 24-hour quit attempt in the past year; **Contemplation:** No quit attempt

Part Three: The use of smoking cessation services

D1. Since last call, have you used any other smoking cessation service in the past year?

1. ☐ Yes 0. ☐ No (Go to part seven)

D2. What smoking cessation service you have used? (Multiple choices)

	No	Yes	(times used)
1. ☐ Telephone counselling	☐	☐	(_________)
2. ☐ Nicotine replacement treatment	☐	☐	(_________)
3. ☐ Face to Face counselling	☐	☐	(_________)
4. ☐ Group discussion	☐	☐	(_________)
5. ☐ Medication treatment	☐	☐	(_________)
6. ☐ Acupuncture treatment	☐	☐	(_________)
7. ☐ Others：_____________________ （Please mark）	☐	☐	(_________)

——— Thank you for your cooperation! This is the end of the questionnaire ———

1 月随访问卷

编码：____________ 日期：______________ 姓名：_________ 联系电话：_________

联络记录：

尝试次数	1	2	3	4	5	6	7
填写人							
日期/时间							
状态							

第一部分：吸烟情况

A1. 您最近7天内有没有吸烟？ 0 ☐ 没有，一口都没有 1 ☐ 有

A1-0-1 您有多长时间一点都没有吸烟？________天

A1-1-1 最近7天内，您大概有几天有吸烟？_____ 天

A1-1-2 过去的30天内，工作日通常一天吸_____ 支；周末吸烟，通常一天吸_____ 支

A2. 您最近7天内有没有使用电子烟？1.☐ 有 0.☐ 没有(跳到A6）

A3. 您使用的电子烟含不含尼古丁成分？1.☐ 有 0.☐ 没有 9. ☐ 不知道

A4. 您最近7天内有没有使用加热烟？1.☐ 有 0.☐ 没有

A5. 除了传统烟、电子烟或者加热烟，您最近7天内还有没有使用其他烟草产品？
0.☐ 没有 (跳到D部分） 1.☐ 有，是__________(请填入烟草产品)

A6. 你最近7天内有使用多少这种烟草产品？共_____次，共______个/支（请在横线上填入具体数字）

A7. 您早晨醒来后多久吸第一支烟？
3.☐ 5 分钟 2.☐ 6-30 分钟内 1.☐ 31-60 分钟内 0.☐ 60 分钟后

A8. 当您身处非吸烟区内，会不会感到难受而忍不住吸烟？ 1. ☐ 会 0. ☐ 不会

A9. 您觉得哪一支烟最难放弃？ 1. ☐早上第一支 0. ☐其它

A10. 您每天抽多少支烟？
0. ☐ 10支或以下 1. ☐ 11-20支 2. ☐ 21-30支 3. ☐ 31支或更多

A11. 当您生病几乎整天卧病在床时还会吸烟吗？ 1. ☐ 会 0. ☐ 不会

A12. 在起床后数小时内，您吸烟次数是否较在其他时间频繁？ 1. ☐ 会 0. ☐ 不会

0To be completed by researcher: Total score: _______
Nicotine Dependency: 1.☐ Mild (0–3) 2.☐ Moderate (4–5) 3.☐ Severe (6 – 10)

第二部分：戒烟史部分：戒烟情况

B1.　自从上次电话之后，有没有认真试过连续24小时以上不吸烟？1 ☐ 有试过　　0 ☐ 没有试过

B2.　自从上次电话之后，你最长有多久没有吸烟? ________天

C1.　您现在有没有打算戒烟？1. ☐ 有　　　　0. ☐ 没有(Pre-contemplation) (跳去 D1)

C2.　您打算从什么时候开始戒烟？(单选)

0. ☐ 我现在已经没有吸烟了 (action) → 确切时间：i)___ 日后 或 ii)___ 月后
1. ☐ 我会在近7天之内戒烟 (preparation/contemplation)
2. ☐ 我会在近1个月之内戒烟 (preparation/contemplation)
3. ☐ 我会在近6个月之内戒烟 (contemplation)
4. ☐ 我会在6个月之后戒烟 (pre-contemplation)
5. ☐ 还没做好决定 (pre-contemplation)

C3.　1. ☐ 已决定的戒烟日期____________________　　0. ☐ 未决定戒烟日期

0 Stage of readiness to quit smoking:

☐ Action　　☐ Preparation#　　☐ Contemplation#　　☐ Pre-contemplation

Preparation: At least one 24-hour quit attempt in the past year; **Contemplation:** No quit attempt

第三部分：戒烟辅导服务使用情况

D1.　自从上次电话之后，你有没有用过任何其他戒烟辅导服务？

1. ☐ 有　　　　0. ☐ 没有（问卷完）

D2.　您使用的是什么服务？（可多选）

	没有	有	（次数）
1. ☐ 电话戒烟辅导	☐	☐	（____）
2. ☐ 尼古丁替代治疗	☐	☐	（____）
3. ☐ 面对面辅导	☐	☐	（____）
4. ☐小组讨论戒烟辅导	☐	☐	（____）
5. ☐ 戒烟药物治疗	☐	☐	（____）
6. ☐ 针灸治疗	☐	☐	（____）
7. ☐ 其他：____________________（请注明）	☐	☐	（____）

——————————多谢您的合作！问卷到此结束——————————

Follow-up Questionnaire: 3 month

Code：________________ Date：_________________Name:____________Contact number: _____________

Contact history:

**Call attempt**	**1**	**2**	**3**	**4**	**5**	**6**	**7**
**Caller**							
**Date/time**							
**Status**							

Part One：Smoking status

A1. Have you smoked in the past 7 days? 1.☐ No, I did not smoke 0.☐ Yes

A1-0-1 How long have you sustained? ______days

A1-1-1.In the past 30 days, How many days do you smoke, Average _____days

A1-1-2.In the past 30 days, How many cigarettes do you smoke on working days? Average ___Cigarettes /day

How many cigarettes do you smoke on weekend? Average ___Cigarettes /day

A2. Do you use e-cigarettes in the past 30 days? 1.☐ Yes 0.☐ No (Go to A6）

A3. Does the e-cigarette you used contain nicotine ingredients?

1.☐ Yes 0.☐ No 9. ☐ Don't know

A4. Do you use heated tobacco products in the past 30 days? 1.☐ Yes 0.☐ No

A5. Do you use any other tobacco products in the past 30 days except for traditional cigarettes, electronic cigarettes or heated cigarettes?

0.☐ No (Go to A9） 1.☐ Yes, It's______（Please fill the category of the tobacco products）

A6. How many such tobacco products have you used in the past 30 days?

A total of _____ times, a total of ______units (please fill in the specific number on the line)\

A7. How soon after waking do you smoke your first cigarete?

3.☐ Within 5 mins 2.☐ 6-30 mins 1.☐ 31-60 mins 0.☐ 60 mins later

A8. Do you find it difficult to refrain from smoking places where it is forbidden? E.g. Church, Library, etc.

1. ☐ Yes 0. ☐ No

A9. Which cigarette would you hate to give up? 1. ☐ The First in the morning 0. ☐ Any other

A10. How many cigarette a day do you smoke?

0. ☐ 10 or less 1. ☐ 11-20 2. ☐ 21-30 3. ☐ 31 or more

A11. Do you smoke more frequently in the morning? 1. ☐ Yes 0. ☐ No

A12. Do you smoke even if you are sick in most of the day? 1. ☐ Yes 0. ☐ No

0To be completed by researcher: Total score: _______

Nicotine Dependency: 1.☐ Mild (0–3) 2.☐ Moderate (4–5) 3.☐ Severe (6 – 10)

Part Two: Quit history

B1. Since last call, how many times have you attempted to quit smoking for more than 24 hours in the past year?
0. ☐ No attempt (Go to D1) 1. ☐ Yes, I attempted________times (Please fill in the specific number on the line)

B2. Since last call, how long have you sustained to no smoke?________days (Please fill in the specific number on the line)

C7. Do you plan to quit smoking now? 1. ☐ Yes 0. ☐ No(Pre-contemplation) (Go to D1)

C8. When do you plan to quit smoking? (Single choice)

0. ☐ I have no smoking now (action)
1. ☐ I will quit smoking within 7 days (preparation/contemplation)
2. ☐ I will quit smoking within 1 month (preparation/contemplation)
3. ☐ I will quit smoking within 6 months (contemplation)
4. ☐ I will quit smoking after 6 months (pre-contemplation)
5. ☐ Have not made a decision yet (pre-contemplation)

The exact time
i)___ days after; or
ii)___ months after.

C9. 1. ☐ Decided smoking cessation date ____________________ 0. ☐ Undecided smoking cessation date

0 Stage of readiness to quit smoking:
☐ Action ☐ Preparation# ☐ Contemplation# ☐ Pre-contemplation
Preparation: At least one 24-hour quit attempt in the past year; **Contemplation:** No quit attempt

Part Three: The use of smoking cessation services

D1. Since last call, have you used any other smoking cessation service in the past year?
1. ☐ Yes 0. ☐ No (Go to part seven)

D2. What smoking cessation service you have used? (Multiple choices)

	No	Yes	(times used)
1. ☐ Telephone counselling	☐	☐	(_________)
2. ☐ Nicotine replacement treatment	☐	☐	(_________)
3. ☐ Face to Face counselling	☐	☐	(_________)
4. ☐ Group discussion	☐	☐	(_________)
5. ☐ Medication treatment	☐	☐	(_________)
6. ☐ Acupuncture treatment	☐	☐	(_________)
7. ☐ Others：_____________________ (Please mark)	☐	☐	(_________)

——— Thank you for your cooperation! This is the end of the questionnaire ———

3 月随访问卷

编码：____________ 日期：______________姓名：_________联系电话：_________

联络记录：

尝试次数	1	2	3	4	5	6	7
填写人							
日期/时间							
状态							

第一部分：吸烟情况

A1. 您最近7天内有没有吸烟？ 0 ☐ 没有，一口都没有 1 ☐ 有

A1-0-1 您有多长时间一点都没有吸烟？________天

A1-1-1 最近7天内，您大概有几天有吸烟？_____ 天

A1-1-2 过去的30天内，工作日通常一天吸_____ 支；周末吸烟，通常一天吸_____ 支

A2. 您最近7天内有没有使用电子烟？1.☐ 有 0.☐ 没有(跳到A6）

A3. 您使用的电子烟含不含尼古丁成分？1.☐ 有 0.☐ 没有 9. ☐ 不知道

A4. 您最近7天内有没有使用加热烟？1.☐ 有 0.☐ 没有

A5. 除了传统烟、电子烟或者加热烟，您最近7天内还有没有使用其他烟草产品？

0.☐ 没有 (跳到D部分） 1.☐ 有，是__________(请填入烟草产品)

A6. 你最近7天内有使用多少这种烟草产品？共_____次，共______个/支（请在横线上填入具体数字）

A7. 您早晨醒来后多久吸第一支煙？

3.☐ 5 分钟 2.☐ 6-30 分钟内 1.☐ 31-60 分钟内 0.☐ 60 分钟后

A8. 当您身处非吸烟区内，会不会感到难受而忍不住吸烟？ 1. ☐ 会 0. ☐ 不会

A9. 您觉得哪一支烟最难放弃？ 1. ☐早上第一支 0. ☐其它

A10. 您每天抽多少支烟？

0. ☐ 10支或以下 1. ☐ 11-20支 2. ☐ 21-30支 3. ☐ 31支或更多

A11. 当您生病几乎整天卧病在床时还会吸烟吗？ 1. ☐ 会 0. ☐ 不会

A12. 在起床后数小时内，您吸烟次数是否较在其他时间频繁？ 1. ☐ 会 0. ☐ 不会

0To be completed by researcher: Total score: _______

Nicotine Dependency: 1.☐ Mild (0–3) 2.☐ Moderate (4–5) 3.☐ Severe (6 – 10)

第二部分：戒烟史部分：戒烟情况

B1. 自从上次电话之后，有没有认真试过连续24小时以上不吸烟？1 ☐ 有试过 0 ☐ 没有试过

B2. 自从上次电话之后，你最长有多久没有吸烟? ________天

C1. 您现在有没有打算戒烟？1. ☐ 有 0. ☐ 没有(Pre-contemplation) (跳去 D1)

C2. 您打算从什么时候开始戒烟？(单选)

0. ☐ 我现在已经没有吸烟了 (action) →
1. ☐ 我会在近7天之内戒烟 (preparation/contemplation)
2. ☐ 我会在近1个月之内戒烟 (preparation/contemplation)
3. ☐ 我会在近6个月之内戒烟 (contemplation)
4. ☐ 我会在6个月之后戒烟 (pre-contemplation)
5. ☐ 还没做好决定 (pre-contemplation)

确切时间：
i)___ 日后 或 ii)___ 月后

C3. 1. ☐ 已决定的戒烟日期____________________ 0. ☐ 未决定戒烟日期

0 Stage of readiness to quit smoking:

☐ Action ☐ Preparation# ☐ Contemplation# ☐ Pre-contemplation

Preparation: At least one 24-hour quit attempt in the past year; **Contemplation:** No quit attempt

第三部分：戒烟辅导服务使用情况

D1. 自从上次电话之后，你有没有用过任何其他戒烟辅导服务？

1. ☐ 有 0. ☐ 没有（问卷完）

D2. 您使用的是什么服务？（可多选）

	没有	有	（次数）
1. ☐ 电话戒烟辅导	☐	☐	(____)
2. ☐ 尼古丁替代治疗	☐	☐	(____)
3. ☐ 面对面辅导	☐	☐	(____)
4. ☐小组讨论戒烟辅导	☐	☐	(____)
5. ☐ 戒烟药物治疗	☐	☐	(____)
6. ☐ 针灸治疗	☐	☐	(____)
7. ☐ 其他：____________________（请注明）	☐	☐	(____)

————————————多谢您的合作！问卷到此结束————————————

Follow-up Questionnaire: 6 month

Code：________________ Date：_________________Name:____________Contact number: _____________

Contact history:

**Call attempt**	**1**	**2**	**3**	**4**	**5**	**6**	**7**
**Caller**							
**Date/time**							
**Status**							

Part One：Smoking status

A1. Have you smoked in the past 7 days? 1.☐ No, I did not smoke 0.☐ Yes

A1-0-1 How long have you sustained? ______days
Biochemical validation appointment:
0. ☐ Refused
1. Time:___________ Date:______________
Venue: ______________________________
Notes:__________________________________

A1-1-1.In the past 30 days, How many days do you smoke, Average _____days
A1-1-2.In the past 30 days, How many cigarettes do you smoke on working days? Average ___Cigarettes /day
How many cigarettes do you smoke on weekend? Average ___Cigarettes /day

A2. Do you use e-cigarettes in the past 30 days? 1.☐ Yes 0.☐ No (Go to A6）

A3. Does the e-cigarette you used contain nicotine ingredients?
1.☐ Yes 0.☐ No 9. ☐ Don't know

A4. Do you use heated tobacco products in the past 30 days? 1.☐ Yes 0.☐ No

A5. Do you use any other tobacco products in the past 30 days except for traditional cigarettes, electronic cigarettes or heated cigarettes?
0.☐ No (Go to A9） 1.☐ Yes, It's______（Please fill the category of the tobacco products）

A6. How many such tobacco products have you used in the past 30 days?
A total of _____ times, a total of ______units (please fill in the specific number on the line)\

A7. How soon after waking do you smoke your first cigarete?
3.☐ Within 5 mins 2.☐ 6-30 mins 1.☐ 31-60 mins 0.☐ 60 mins later

A8. Do you find it difficult to refrain from smoking places where it is forbidden? E.g. Church, Library, etc.
1. ☐ Yes 0. ☐ No

A9. Which cigarette would you hate to give up? 1. ☐ The First in the morning 0. ☐ Any other

A10. How many cigarette a day do you smoke?
0. ☐ 10 or less 1. ☐ 11-20 2. ☐ 21-30 3. ☐ 31 or more

A11. Do you smoke more frequently in the morning? 1. ☐ Yes 0. ☐ No

A12. Do you smoke even if you are sick in most of the day? 1. ☐ Yes 0. ☐ No

0To be completed by researcher: Total score: _______
Nicotine Dependency: 1.☐ Mild (0–3) 2.☐ Moderate (4–5) 3.☐ Severe (6 – 10)

Part Two: Quit history

B1. Since last call, how many times have you attempted to quit smoking for more than 24 hours in the past year?
0. ☐ No attempt (Go to D1) 1. ☐ Yes, I attempted________times (Please fill in the specific number on the line)

B2. Since last call, how long have you sustained to no smoke?________days (Please fill in the specific number on the line)

C10. Do you plan to quit smoking now? 1. ☐ Yes 0. ☐ No(Pre-contemplation) (Go to D1)

C11. When do you plan to quit smoking? (Single choice)

0. ☐ I have no smoking now (action)
1. ☐ I will quit smoking within 7 days (preparation/contemplation)
2. ☐ I will quit smoking within 1 month (preparation/contemplation)
3. ☐ I will quit smoking within 6 months (contemplation)
4. ☐ I will quit smoking after 6 months (pre-contemplation)
5. ☐ Have not made a decision yet (pre-contemplation)

The exact time
i)___ days after; or
ii)___ months after.

C12. 1. ☐ Decided smoking cessation date ____________________ 0. ☐ Undecided smoking cessation date

0 Stage of readiness to quit smoking:
☐ Action ☐ Preparation# ☐ Contemplation# ☐ Pre-contemplation
Preparation: At least one 24-hour quit attempt in the past year; **Contemplation:** No quit attempt

Part Three: The use of smoking cessation services

D1. Since last call, have you used any other smoking cessation service in the past year?
1. ☐ Yes 0. ☐ No (Go to part seven)

D2. What smoking cessation service you have used? (Multiple choices)

	No	Yes	(times used)
1. ☐ Telephone counselling	☐	☐	(_________)
2. ☐ Nicotine replacement treatment	☐	☐	(_________)
3. ☐ Face to Face counselling	☐	☐	(_________)
4. ☐ Group discussion	☐	☐	(_________)
5. ☐ Medication treatment	☐	☐	(_________)
6. ☐ Acupuncture treatment	☐	☐	(_________)
7. ☐ Others：_____________________ （Please mark）	☐	☐	(_________)

Thank you for your cooperation! This is the end of the questionnaire

6 月随访问卷

编码：____________ 日期：______________姓名：_________联系电话：_________

联络记录：

尝试次数	1	2	3	4	5	6	7
填写人							
日期/时间							
状态							

第一部分：吸烟情况

A1. 您最近7天内有没有吸烟？ 0 ☐ 没有，一口都没有 1 ☐ 有

A1-0-1 您有多长时间一点都没有吸烟？________天

预约生化检测：

0. ☐ 拒绝

1.预约时间：________ 日期：________

预约地点：________________________

备注：____________________________

A1-1-1 最近7天内，您大概有几天有吸烟？_____ 天

A1-1-2 过去的30天内，工作日通常一天吸_____ 支；周末吸烟，通常一天吸_____ 支

A2. 您最近7天内有没有使用电子烟？1.☐ 有 0.☐ 没有(跳到A6）

A3. 您使用的电子烟含不含尼古丁成分？1.☐ 有 0.☐ 没有 9. ☐ 不知道

A4. 您最近7天内有没有使用加热烟？1.☐ 有 0.☐ 没有

A5. 除了传统烟、电子烟或者加热烟，您最近7天内还有没有使用其他烟草产品？
0.☐ 没有 (跳到D部分） 1.☐ 有，是__________（请填入烟草产品）

A6. 你最近7天内有使用多少这种烟草产品？共_____次，共______个/支（请在横线上填入具体数字）

A7. 您早晨醒来后多久吸第一支煙？
3.☐ 5 分钟 2.☐ 6-30 分钟内 1.☐ 31-60 分钟内 0.☐ 60 分钟后

A8. 当您身处非吸烟区内，会不会感到难受而忍不住吸烟？ 1. ☐ 会 0. ☐ 不会
A9. 您觉得哪一支烟最难放弃？ 1. ☐早上第一支 0. ☐其它
A10. 您每天抽多少支烟？
0. ☐ 10支或以下 1. ☐ 11-20支 2. ☐ 21-30支 3. ☐ 31支或更多
A11. 当您生病几乎整天卧病在床时还会吸烟吗？ 1. ☐ 会 0. ☐ 不会
A12. 在起床后数小时内，您吸烟次数是否较在其他时间频繁？ 1. ☐ 会 0. ☐ 不会

0To be completed by researcher: Total score: _______
Nicotine Dependency: 1.☐ Mild (0–3) 2.☐ Moderate (4–5) 3.☐ Severe (6 – 10)

第二部分：戒烟史部分：戒烟情况

B1. 自从上次电话之后，有没有认真试过连续24小时以上不吸烟？1 ☐ 有试过 0 ☐ 没有试过
B2. 自从上次电话之后，你最长有多久没有吸烟？ ________天
C1. 您现在有没有打算戒烟？1. ☐ 有 0. ☐ 没有(Pre-contemplation) (跳去 D1)
C2. 您打算从什么时候开始戒烟？(单选)
0. ☐ 我现在已经没有吸烟了 (action) → 确切时间：i)___ 日后 或 ii)___ 月后
1. ☐ 我会在近7天之内戒烟 (preparation/contemplation)
2. ☐ 我会在近1个月之内戒烟 (preparation/contemplation)
3. ☐ 我会在近6个月之内戒烟 (contemplation)
4. ☐ 我会在6个月之后戒烟 (pre-contemplation)
5. ☐ 还没做好决定 (pre-contemplation)
C3. 1. ☐ 已决定的戒烟日期___________________ 0. ☐ 未决定戒烟日期

0 Stage of readiness to quit smoking:
☐ Action ☐ Preparation# ☐ Contemplation# ☐ Pre-contemplation
Preparation: At least one 24-hour quit attempt in the past year; **Contemplation:** No quit attempt

第三部分：戒烟辅导服务使用情况

D1. 自从上次电话之后，你有没有用过任何其他戒烟辅导服务？
1. ☐ 有 0. ☐ 没有（问卷完）
D2. 您使用的是什么服务？（可多选）

	没有	有	（次数）
1. ☐ 电话戒烟辅导	☐	☐	（____）
2. ☐ 尼古丁替代治疗	☐	☐	（____）
3. ☐ 面对面辅导	☐	☐	（____）
4. ☐小组讨论戒烟辅导	☐	☐	（____）
5. ☐ 戒烟药物治疗	☐	☐	（____）
6. ☐ 针灸治疗	☐	☐	（____）
7. ☐ 其他：____________________（请注明）	☐	☐	（____）

——————————多谢您的合作！问卷到此结束——————————
