# Supplementary material for: Effectiveness of a video-based smoking cessation intervention focusing on maternal and child health in promoting quitting among expectant fathers in China: A randomized controlled trial
Source: PLoS Med. 2020 Sep 29;17(9):e1003355. doi: 10.1371/journal.pmed.1003355 (PMC7523971; doi:10.1371/journal.pmed.1003355)
Supplement: S5 Text — (S5A) Multiple imputation. (S5B) GEE model. (S5C) Sensitivity analysis. GEE, generalized estimating equation (DOCX) [file pmed.1003355.s006.docx]

## S5 Text. Details of statistical analysis method.

**S5A Text. Multiple imputation**

The multiple imputations were performed with ***mice*** package v2.25 using the Markov Chain Monte Carlo method to generate 30 data sets with the imputed missing data [1-3]. The imputation model included socio-demographic characteristics and smoking-related variables at baseline and outcomes at all follow-ups, including age, hospital, employment status, annual income level, hospital, father’s parity, level of nicotine dependence, level of readiness to quit, smoking self-efficacy, validated 7-day PPA, self-reported 7-day PPA, and level of readiness to quit, with two-way interactions among categorical predictors. These variables were also included in the model to reduce bias and incompatibility. Regression parameters and the corresponding SEs of all the imputed data sets were pooled using Rubin’s rules [4].

**S5B Text. Generalized Estimating Equation Model**

The Generalized Estimating Equations were performed with ***geepack*** package [5], with adjustment of age, hospital, employment status, annual income level, hospital, father’s parity, level of nicotine dependence, level of readiness to quit, and smoking self-efficacy at baseline.

The GEE was firstly performed for the estimation of primary outcome at 6 months follow-up and secondary outcomes at each follow-up. The primary outcome (validated 7-day PPA) was only measured at baseline and 6 months follow-up. Considering all participants were smokers at baseline, a repeated measure can not be performed due to the matrix at baseline was singular. Given that the repeated measure was further performed using the Covariance estimator of Robust estimator and AutoRegressive Order 1 (AR1) for the self-reported 7-day PPA by excluding the baseline values and for the level of readiness to quit at all time points, respectively.

**S5C Text. Sensitivity analysis**

*Intention-to-treat method*

As required by the Russell Standard, in smoking cessation trials, participants who do not report their smoking status at follow-up are often assumed to be smoking [6,7]. In the Primary analysis using the ITT method, all participants were included in the data analysis. The missing values of outcome variables were replaced by assuming subjects lost to follow-up to be active smokers with no changes in their habit. Then the missing values of the demographic and smoking profiles at baseline were handled using multiple imputations by including age, hospital, employment status, annual income level, hospital, father’s parity, level of nicotine dependence, level of readiness to quit, and smoking self-efficacy, and outcomes replaced by baseline values. Then the GEE model was performed using the imputed dataset.

*Pattern-mixture model with multiple imputation (PMM-MI)*

In the PPM-MI analysis, the pattern parameter estimates were firstly generated [8]. The missing outcomes were imputed in the covariates using the pattern specified by the identifying restriction chosen with the assumption of NMAR. Finally, estimation was fitted using the imputed dataset incorporates a full group-by-time interaction at each follow-up and combined using Rubin’s rules [9,10].

*Completed case*

All missing values were excluded in the estimation.

## References

[1]. Van Buuren S, Groothuis-Oudshoorn K, Robitzsch A, at al. mice: Multivariate Imputation by Chained Equations in R. 2015. https://cran.r-project.org/web/packages/mice/index.html Accessed on 17 April 2017

[2]. Jakobsen JC, Gluud C, Wetterslev J, et al. When and how should multiple imputation be used for handling missing data in randomised clinical trials - a practical guide with flowcharts. BMC Med Res Methodol 2017; 17: 162.

[3]. White IR, Royston P, Wood AM. Multiple imputation using chained equations: Issues and guidance for practice. Stat Med. 2011;30(4):377‐399. doi:10.1002/sim.4067

[4]. Schafer JL. Analysis of Incomplete Multivariate Data 1st ed. New York: Chapman & Hall/CRC; 1997

[5]. Halekoh U, Højsgaard S, Yan J. The R package geepack for generalized estimating equations. J Stat Softw 2006; 15(2): 1-11.

[6]. West R., Hajek P., Stead L., Stapleton J. Outcome criteria in smoking cessation trials: the need for a common standard. Addiction 2005; 100: 299–303.

[7]. Jackson D, White IR, Mason D, Sutton S. A general method for handling missing binary outcome data in randomized controlled trials. Addiction. 2014;109:1986–93.

[8]. Bishop, B. (2017). Examining Random-Coeffcient Pattern-Mixture Models for Longitudinal Data with Informative Dropout (Doctoral dissertation, The Ohio State University).

[9]. Iddrisu AK, Gumedze F. An application of a pattern-mixture model with multiple imputation for the analysis of longitudinal trials with protocol deviations. BMC Med Res Methodol. 2019;19(1):10.

[10]. Bunouf, P., Molenberghs, G., Grouin, J. M., & Thijs, H. (2015). A SAS program combining R functionalities to implement pattern-mixture models. [6]. Baker TB, Piper ME, Schlam TR, et al. Are tobacco dependence and withdrawal related amongst heavy smokers? Relevance to conceptualizations of dependence. J Abnorm Psychol 2012;121:909-21.
