# Supplementary material for: Effectiveness of a video-based smoking cessation intervention focusing on maternal and child health in promoting quitting among expectant fathers in China: A randomized controlled trial
Source: PLoS Med. 2020 Sep 29;17(9):e1003355. doi: 10.1371/journal.pmed.1003355 (PMC7523971; doi:10.1371/journal.pmed.1003355)
Supplement: S6 Text — (DOCX) [file pmed.1003355.s007.docx]

**S6 Text. Finding in the subgroup analysis.**

The results of our subgroup analysis suggested that the video intervention had no effect on smoking expectant fathers with moderate or higher levels of nicotine dependency at baseline. Previous research shows that nicotine abstinence leads to withdrawal symptoms, especially for smokers with higher levels of nicotine dependence.[6] Discomfort from withdrawal symptoms can undermine the determination to quit smoking [1]. In this study, the video intervention only focused on the hazards of smoking and did not address strategies to manage withdrawal symptoms. Fathers with moderate or high nicotine dependence may not have known how to manage their withdrawal symptoms. Discomfort associated with these symptoms could have been a barrier to successful quitting, despite the motivation to do so. This is probably why the video intervention was more beneficial for fathers with low nicotine dependence than for those with moderate or high nicotine dependence. In addition, the subgroup analysis results demonstrated that fathers with no intention to quit at baseline reported higher odds of quitting at 6 months than those intending to quit at baseline. These results suggest that the video intervention may have a stronger effect on motivating smokers with no initial intention to quit to attempt to quit smoking. Previous research shows that one of the main reasons that Chinese smoking expectant fathers are unwilling to quit smoking is a lack of awareness of the importance of quitting [2]. Therefore, the use of videos to increase their knowledge of smoking hazards may change their perceptions about smoking and motivate them to quit. Individuals intending to quit at baseline may already have been aware of the importance of quitting [3]. Previous studies indicate that smokers who intend to quit require cessation support (e.g., a concrete quit plan and relapse prevention skills) that goes beyond simply increasing knowledge of smoking hazards. Therefore, our intervention may be less effective in promoting quitting among this group of smokers.

## References

[1]. Powell J, Dawkins L, West R, et al. Relapse to smoking during unaided cessation: clinical, cognitive and motivational predictors. Psychopharmacology 2010;212:537-49.

[2]. McCaul KD, Hockemeyer JR, Johnson RJ, et al. Motivation to quit using cigarettes: a review. Addict Behav 2006;31:42-56.

[3]. Feng G, Jiang Y, Li Q, et al. Individual-level factors associated with intentions to quit smoking among adult smokers in six cities of China: findings from the ITC China Survey. Tob Control 2010;19 Suppl 2:i6-11.
