# Supplementary material for: Effectiveness of a video-based smoking cessation intervention focusing on maternal and child health in promoting quitting among expectant fathers in China: A randomized controlled trial
Source: PLoS Med. 2020 Sep 29;17(9):e1003355. doi: 10.1371/journal.pmed.1003355 (PMC7523971; doi:10.1371/journal.pmed.1003355)
Supplement: S1 Table — (DOCX) [file pmed.1003355.s008.docx]

S2 Table. **Repeated measure for secondary outcomes.**

| Variables | Video vs Text | | |  | | Video vs Control | | |  | | Text vs Control | | | |
| --- | --- | --- | --- | --- | --- | --- | --- | --- | --- | --- | --- | --- | --- | --- |
|  | Adjusted OR (95% CI)^a^ | P value |  | | Adjusted OR (95% CI)^a^ | | P value |  | | Adjusted OR (95% CI)^a^ | | P value |  |  |
| Self-reported 7-day point prevalence of abstinence | | | | | | | | | | | | | |  |
|  | 1.45(1.02-2.04) | 0.02 |  | | 2.38(1.70-3.33) | | <0.001 |  | | 1.47(1.05-2.08) | | 0.02 |  |  |
| Readiness to quit within 30-day^c^ | | | | | | | | | | | | | |  |
|  | 1.35(0.92-1.98) | 0.12 |  | | 1.75(1.16-2.79) | | 0.007 |  | | 1.30(1.12-1.96) | | 0.01 |  |  |

Abbreviation: OR, Odds Ratio; CI, Confidence Interval;

^a^ Participants lost to follow-up were assumed to be active smokers with no changes in their habits at baseline. Adjusted estimates from the GEE model with repeated measure adjusted for age, hospital, employment status, annual income level, hospital, father’s parity, level of nicotine dependence, level of readiness to quit, and smoking self-efficacy at baseline.
