# Supplementary material for: Effectiveness of a video-based smoking cessation intervention focusing on maternal and child health in promoting quitting among expectant fathers in China: A randomized controlled trial
Source: PLoS Med. 2020 Sep 29;17(9):e1003355. doi: 10.1371/journal.pmed.1003355 (PMC7523971; doi:10.1371/journal.pmed.1003355)
Supplement: S2 Table — (DOCX) [file pmed.1003355.s009.docx]

## **S2 Table. Demographic and smoking characteristics between participants completed and lost to follow-up at 6-month.**

|  | No. (%)^a^ | |  |
| --- | --- | --- | --- |
| Variables | Completed follow-up (n=815) | Lost to follow-up (n=208) | P value |
| Research arm |  |  | < 0.001 |
| Video group | 284/333(85.3) | 49/333(14.7) |  |
| Text group | 270/322(83.9) | 52/322(16.1) |  |
| Control group | 261/468(70.9) | 107/368(29.1) |  |
| Subjects resources |  |  | 0.07 |
| Hospital A | 210(25.8) | 38(18.3) |  |
| Hospital B | 369(45.3) | 100(48.1) |  |
| Hospital C | 236(29.0) | 70(33.7) |  |
| Age, mean (SD), year | 31. 9(5.2) | 32.6(5.9) | 0.14 |
| Employment status |  |  | 0.35 |
| Unemployed | 575(75.0) | 151(74.4) |  |
| Self-employment | 82(10.7) | 28(13.8) |  |
| Employed | 110(14.3) | 24(11.8) |  |
| Education level |  |  | 0.09 |
| Primary school or below | 13(1.6) | 4(1.9) |  |
| Middle school | 256(31.7) | 49(23.8) |  |
| College/university or above | 539(66.7) | 153(74.3) |  |
| Annual Family income (CNY) ^a^ |  |  | 0.06 |
| ¥ 9999 or below | 119(14.9) | 18(9.0) |  |
| ¥ 10000-49999 | 141(17.6) | 26(12.9) |  |
| ¥ 50000-99999 | 256(32.0) | 74(36.8) |  |
| ¥ 100,000-199,999 | 135(16.9) | 37(18.4) |  |
| ¥ 200,000or above | 149(18.6) | 46(22.9) |  |
| First time to be expectant father |  |  | 0.83 |
| Yes | 424(52.0) | 110(52.9) |  |
| No | 391(48.0) | 98(47.1) |  |
| Living with smoking partners |  |  | 0.32 |
| Yes | 5(0.6) | 0(0) |  |
| No | 810(99.4) | 208(100) |  |
| Monthly regular alcohol use |  |  | 0.16 |
| No | 304(37.3) | 86(41.3) |  |
| Yes | 511(62.7) | 122(58.7) |  |
| Regular activity at least 1hr/week |  |  | 0.42 |
| Yes | 271(33.3) | 67(32.2) |  |
| No | 544(66.7) | 141(67.8) |  |
| Physical health status (SF-12-PCS), mean (SD) | 52.9(4.00) | 52.9(3.9) | 0.86 |
| Mental health status (SF-12-MCS), mean (SD) | 52.7(6.8) | 53.5(6.3) | 0.16 |
| Daily cigarette consumption, mean (SD) | 9.5(6.1) | 9.6(6.0) |  |
| Duel use of e-cigarette | 3(0.4) | 2(1.0) | 0.27 |
| Duel use of IQOS | 2(0.2) | 0(0.0) | 0.64 |
| Nicotine Dependence level (FTND) |  |  | 0.50 |
| Low dependence (0-3 Fagerstrom score) | 537(66.2) | 136(66.0) |  |
| Moderate dependence (4-5 Fagerstrom score) | 196(24.2) | 45(21.8) |  |
| High dependence (6-10 Fagerstrom score) | 78(9.6) | 25(12.1) |  |
| Readiness to quit within 30-day |  |  | 0.20 |
| Yes | 78(9.6) | 14(6.7) |  |
| No | 737(90.4) | 194(93.3) |  |
| Years of regular tobacco use | 14.2(5.9) | 14.3(6.0) | 0.86 |
| Quit attempt for 24 hours within one year |  |  | 0.02 |
| Yes | 212(26.0) | 39(18.8) |  |
| No | 603(74.0) | 169(81.3) |  |
| Latest quit attempt date |  |  | 0.25 |
| Within recent one month | 28(13.2) | 3(7.7) |  |
| More than one month ago | 184(86.8) | 36(92.3) |  |
| Seek for smoking cessation service |  |  | 0.80 |
| Yes | 1(0.1) | 0(0) |  |
| No | 816(99.9) | 208(100) |  |
| Smoking self-efficacy scored by SEQ-12 (12-60), mean (SD) | 31.2(10.6) | 33.3(10.4) | 0.84 |

Abbreviations: SF-12, 12-Item Short-Form Survey; PCS, Physical Health Status; MCS, Mental Health Status; FTND, Fagerström Test of Nicotine Dependence; SEQ-12, Smoking Self-efficacy Questionnaire.

^a^ Sample sizes varied because of missing data on some variables.

^b^ ¥/CNY represents China Yuan, US$1.00 = ¥ 6.7.
