# Supplementary figures and images for: Effectiveness of a video-based smoking cessation intervention focusing on maternal and child health in promoting quitting among expectant fathers in China: A randomized controlled trial
Source: PLoS Med. 2020 Sep 29;17(9):e1003355. doi: 10.1371/journal.pmed.1003355 (PMC7523971; doi:10.1371/journal.pmed.1003355)

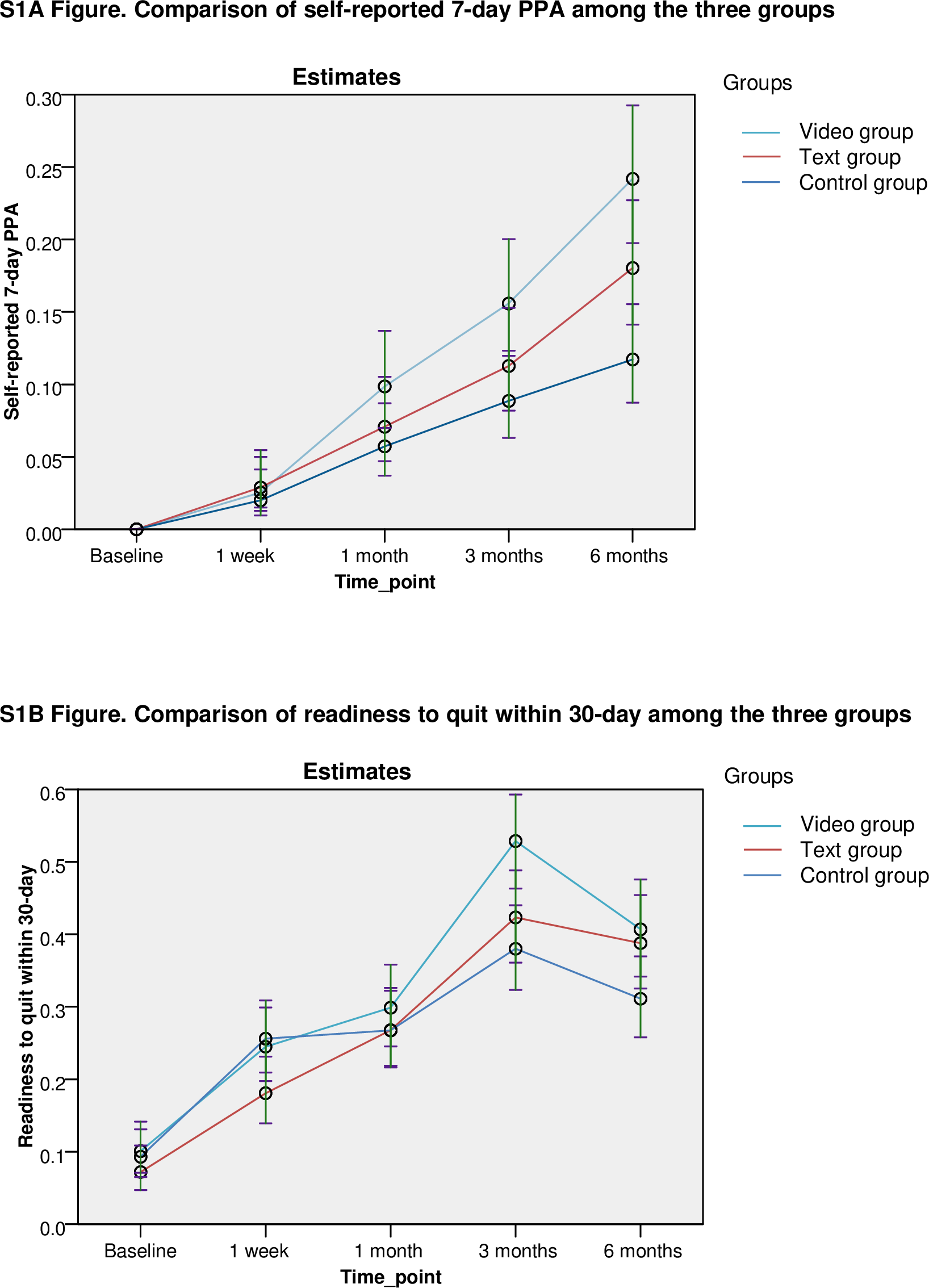

Supplement: S1 Fig — (S1A) Comparison of self-reported 7-day PPA among the 3 groups. (S1B) Comparison of readiness to quit within 30 days among the 3 groups. PPA, point prevalence of abstinence. (TIF) [file pmed.1003355.s010.tif]
